# On the Entropy-Based Localization of Inequality in Probability Distributions

**DOI:** 10.3390/e27090945

**Published:** 2025-09-10

**Authors:** Rajeev Rajaram, Nathan Ritchey, Brian Castellani

**Affiliations:** 1 Department of Mathematical Sciences, Kent State University, Kent, OH 44242, USA; nritche2@kent.edu; 2 Durham Research Methods Centre, Durham University, Stockton Road, Durham DH1 3LE, UK; brian.c.castellani@durham.ac.uk

**Keywords:** Shannon entropy, degree of uniformity, Hahn decomposition

## Abstract

We present a novel method for localizing inequality within probability distributions by applying a recursive Hahn decomposition to the degree of uniformity—a measure derived from the exponential of Shannon entropy. This approach partitions the probability space into disjoint regions exhibiting progressively sharper deviations from uniformity, enabling structural insights into how and where inequality is concentrated. To demonstrate its broad applicability, we apply the method to both standard and contextualized systems: the discrete binomial and continuous exponential distributions serve as canonical cases, while two hypothetical examples illustrate domain-specific applications. In the first, we analyze localized risk concentrations in disease contraction data, revealing targeted zones of epidemiological disparity. In the second, we uncover stress localization in a non-uniformly loaded beam, demonstrating the method’s relevance to physical systems with spatial heterogeneity. This decomposition reveals aspects of structural disparity that are often obscured by scalar summaries. The resulting recursive tree offers a multi-scale representation of informational non-uniformity, capturing the emergence and localization of inequality across the distribution. The framework may have implications for understanding entropy localization, transitions in informational structure, and the dynamics of heterogeneous systems.

## 1. Introduction

A defining characteristic of probability distributions is the presence of inherent inequality; some outcomes occur with higher probability than others. This asymmetry manifests in the distribution’s shape and is typically interpreted using global measures like the center and spread. However, a more refined understanding requires both quantification and localization of this inequality. Quantification, which has been the primary focus of our previous work [1,2,3,4], provides a rigorous, scale-free means of measuring how uniformly probability is distributed either across the entire space or within specific measurable subsets based solely on their inherent uncertainty. This enables comparisons of inequality both within a single distribution and across different distributions. The present paper, by contrast, centers on localizing the systematic identification of where in the probability space inequality is most pronounced. Without localization, global measures risk masking critical disparities within the distribution. By applying the Hahn decomposition theorem to the degree of uniformity measure, we introduce a recursive, principled framework that decomposes the distribution into disjoint regions of varying inequality. This shift in focus from quantifying how much inequality exists to revealing where it resides is essential for applications that demand both interpretability and targeted insights, such as diagnostics in statistical modeling, fairness analysis, or information-driven decision-making.

To situate our contribution within the broader literature, we briefly review prior entropy-based approaches to diversity and complexity. Entropy-based measures have long served as foundational tools for quantifying diversity, inequality, and complexity across disciplines. Shannon entropy captures the uncertainty of a distribution, while Hill numbers—widely used in ecology and information theory—translate entropy into effective counts of equally likely outcomes. These Hill numbers are mathematically linked to Renyi entropies; the Hill number of order *q* is the exponential of Renyi entropy of the same order, offering a unified framework for interpreting diversity and concentration.

Building on this lineage, LMC-type statistical complexity measures [5] combine entropy with disequilibrium to quantify non-uniformity in probability densities. Recent refinements [6] use differential-escort transformations to ensure monotonicity of these complexity metrics, particularly in systems governed by the power-law or non-extensive statistics. However, these approaches remain scalar in nature, offering global summaries of complexity without spatial or hierarchical localization.

In contrast, our contribution introduces a recursive Hahn decomposition of an entropy-derived signed measure, enabling structural localization of inequality within the probability space. This method partitions the space into disjoint subsets with monotonic ordering of uniformity, explicitly identifies null sets, and constructs a ternary tree that reveals the geometry of inequality across scales. By shifting from scalar quantification to recursive decomposition, we offer a new lens for analyzing distributions where localized disparities matter.

### 1.1. Ideas So Far

Uniform distributions, by their nature, exhibit a mathematical diversity that aligns directly with the support of the distribution. For discrete uniform distributions supported on {1,…,K}, the diversity ^1^*D*, defined as the exponential of Shannon entropy and corresponding to the Hill number of order q=1, is precisely *K*. Similarly, for continuous uniform distributions over the interval (a,b), the diversity ^1^*D* is equal to (b−a). Any departure from uniformity results in a reduced diversity when compared to a uniform distribution with the same support.

The concept of diversity ^1^*D* [7,8,9,10] carries an intuitive interpretation: one can conceptually transform the original non-uniform distribution into a Shannon Equivalent Equi-probable (SEE) uniform distribution, where the size of the support matches ^1^*D*. Although this equivalence is abstract, it proves valuable for visualizing the notion of diversity.

Diversity can also be analyzed in the context of sub-components of a distribution, as discussed in [1,2]. Specifically, a subset *P* with cumulative probability $c_{P}=\sum_{i\in P}p_{i}$ and Shannon diversity $D_{P}=\exp\left(H_{P}\right)$, where $H_{P}=-\sum_{i\in P}\frac{p_{i}}{c_{P}}\ln\left(\frac{p_{i}}{c_{P}}\right)$ is the entropy of the part *P*, can be evaluated using the ratio $D_{P}/c_{P}$ to assess its degree of uniformity.

The term *Shannon Equivalent* is used to emphasize that the original segment or the entire distribution thereof shares the same conditional Shannon entropy as its SEE counterpart. We have selected the exponential of Shannon entropy, also known as *mathematical diversity* ^1^*D* (or DP1cP if it is a part *P* and not the whole), to quantify the degree of inequality present in distributions, as outlined in [1,2]. This choice is primarily motivated by the fact that, among the Hill numbers *^q^D*, where *q* is a parameter governing various diversity measures, the value corresponding to q=1 strikes an equal balance between the richness and evenness of the distribution. Furthermore, Shannon entropy is widely recognized as a fundamental measure of probabilistic, or information-theoretic uncertainty in a distribution.

Rather than directly quantifying the inequality of the original part or whole, which may not exhibit uniformity, the abstract SEE equivalent enables us to assess and compare the degree of uniformity on a common ground. This is because the redrawn SEE equivalents are (a) uniform distributions and (b) possess the same entropic uncertainty as the original part or whole from which they were derived. The uniformity inherent in the distribution of SEE parts (albeit abstract) makes it significantly easier to compare and quantify their degree of uniformity.

Much of our previous work [1,2,3,4] was focused on the *quantification* of inequality in probability distributions. This was an important first step, where given a probability distribution or a part (or parts from different distributions), we aimed to compute and compare the degree of inequality of the given part (s) by computing $\frac{D_{P}}{c_{P}}$. We use the terms *degree of uniformity* and *degree of inequality* interchangeably in this paper since we have shown these two concepts to be equivalent in [1,2,3,4].

To quickly reiterate the idea, if we assume that $P_{1}$ and $P_{2}$ are two parts with $\frac{\frac{D_{P_{1}}}{c_{P_{1}}}}{\frac{D_{P_{2}}}{c_{P_{2}}}}=R>1$, say, then this means that, with respect to the entropy-derived measure used in this paper, the degree of uniformity defined as D/c-the part $P_{1}$ is *R* times more uniformly distributed than $P_{2}$. We emphasize that this interpretation is specific to the Shannon entropy framework adopted here. Other complementary measures of uniformity exist, such as LMC-type statistical complexity metrics [5,6], which combine entropy with disequilibrium to quantify non-uniformity from a different perspective. Our method focuses on structural localization of inequality using recursive Hahn decomposition, whereas LMC complexity offers a scalar summary of global non-uniformity. To bring in the idea of inequality here, we also note that, equivalently, this means that $P_{2}$ is *R* times less uniformly distributed compared to $P_{1}$. Hence, $P_{2}$ is *R* times more concentrated than $P_{1}$ as well. This is also clear by noting that $\frac{\frac{c_{P_{2}}}{D_{P_{2}}}}{\frac{c_{P_{1}}}{D_{P_{1}}}}=\frac{\frac{D_{P_{1}}}{c_{P_{1}}}}{\frac{D_{P_{2}}}{c_{P_{2}}}}=R>1$. In other words, a larger ratio of $\frac{c_{P}}{D_{P}}$ means a higher concentration of probability (or frequency) over the part *P*.

Given this explanation, we use the terms *degree of uniformity* and *degree of inequality* interchangeably for the ratio $\frac{D_{P}}{c_{P}}$ for a given part *P*, with the caveat that the two concepts are inverses of each other, i.e., a higher degree of uniformity means a lower degree of inequality and vice-versa. This makes intuitive sense, as more uniformity for a part *P* means that the mass is more spread out in an equivalent equiprobable form. Hence, its opposite concept, i.e., inequality, should be that the part *P* is more concentrated rather than spread out, in its equivalent equiprobable form.

### 1.2. Potential Applications

The computation and comparison of inequality or uniformity within parts of a distribution have significant applications across various fields. In structural engineering, analyzing stress or strain distribution along beams or surfaces is crucial to identify regions prone to failure or excessive loading, ensuring structural safety and efficiency. In economics, measuring income inequality across different regions or demographic groups helps policymakers design equitable taxation and social welfare programs. In environmental science, the study of pollutant concentration distributions across ecosystems aids in identifying hotspots of contamination and planning remediation efforts. Similarly, in health care, the uniformity of drug distribution in the bloodstream or across a target organ can influence the effectiveness of treatment, such as chemotherapy or targeted drug delivery. In manufacturing, uniformity in thickness or material properties in products like films or coatings ensures quality and durability, while inequality could indicate defects or inefficiencies. In data communication, assessing the distribution of traffic in networks helps optimize load balancing and prevent bottlenecks. In physics, comparing energy or particle density distributions within systems can reveal insights into underlying processes, such as in plasma physics or astrophysics. In education, analyzing the distribution of test scores across schools or regions can highlight disparities in educational access or effectiveness. Across all these domains, understanding and comparing inequality or uniformity of distributions is integral to decision-making and optimization.

Entropy-based techniques have previously been applied to epidemiological modeling and mechanical stress analysis, often through scalar metrics such as Shannon entropy or LMC-type complexity measures [5,6]. Our recursive decomposition offers a structural alternative that localizes inequality and reveals null regions, potentially enhancing interpretability in these domains.

### 1.3. New in This Paper

Given that we can now precisely measure the degree of inequality of a probability distribution or its parts using the ratio $\frac{D_{P}}{c_{P}}$ for the part *P*, and also the fact that the degree of uniformity of a part is the weighted geometric mean of the degrees of uniformity of its sub-parts as seen in Theorem 2, we pondered on the following two questions:

1. Is there a way to systematically locate inequality in a given distribution, i.e., divide a given probability distribution into subsets with monotonically decreasing degrees of uniformity (or equivalently increasing degrees of inequality)?
2. Can we do this in a recursive way and maintain the nested ordering of degrees of inequality of parts? In other words, measurable subsets of two different measurable sets $S_{1}\subset P_{1}$ and $S_{2}\subset P_{2}$ with the ordering $\frac{D_{P_{1}}}{c_{P_{1}}}>\frac{D_{P_{2}}}{c_{P_{2}}}$ should also follow the same ordering of degree of inequality, i.e., $\frac{D_{S_{1}}}{c_{S_{1}}}>\frac{D_{S_{2}}}{c_{S_{2}}}$ (here $\frac{D_{P}}{c_{P}}$ is the degree of uniformity of the part *P*).

We emphasize the importance of preserving the ordering of inequality when subdividing a distribution. While one might use arbitrary cutoff points such as visual cues from probability values to define subsets, this does not guarantee *monotonicity* in the degree of inequality. In contrast, our method ensures that the monotonicity of degree of inequality is preserved, i.e., subsets of $P_{1}$ and $P_{2}$ with the ordering $\frac{D_{P_{1}}}{c_{P_{1}}}>\frac{D_{P_{2}}}{c_{P_{2}}}$ will automatically follow the same ordering of degree of inequality like we mentioned in the second point above.

Maintaining the monotonicity of the degree of inequality during subdivision is crucial for revealing the granularity of its localization. The method introduced in this paper enables a recursive partitioning of the probability space, where the cutoff points are determined by recursively employing the Hahn decomposition of the degree of uniformity measure. This approach ensures that each step refines the space into subsets with a well-defined and preserved ordering of inequality, derived from mathematical diversity, a concept rooted in entropy. The process continues until no further meaningful subdivisions arise, all while maintaining the hierarchy of inequality across levels. This allows for a coherent, scalable, and information-theoretically grounded localization of inequality.

The paper is organized as follows: In Section 2.1, we briefly introduce the idea of a general signed measure and its Hahn decomposition. For a detailed discussion on measure theory, we refer to [11]. In Section 2.2, we recall some of the important background material related to the idea of mathematical diversity that is relevant to this paper. In Section 3, we state and prove the main results of this paper related to the Hahn decomposition of the degree of uniformity measure. In Section 4, we show some applications of our method to specific examples. In Section 5, we conclude with some observations related to the main results in the paper.

## 2. Preliminaries

This section consolidates all foundational concepts required for the main results. We begin with signed measures and Hahn decomposition and proceed to diversity indices.

### 2.1. Signed Measures and Hahn Decomposition

A *signed measure* is an extension of the mathematical concept of measure, allowing values to be both positive and negative. Unlike a traditional measure, which assigns non-negative values to sets in a sigma-algebra, a signed measure can assign negative values, enabling it to model a broader range of phenomena. Key to understanding a signed measure are the notions of positive, negative, and null sets. A positive set is one where the signed measure assigns a non-negative value to every measurable subset, while a negative set has the signed measure assigning non-positive values to all measurable subsets. A null set, on the other hand, has a signed measure of zero for all its measurable subsets, making it distinct from a set of measure zero, which might still carry subsets with non-zero measures in the signed measure framework. The Hahn decomposition theorem formalizes the fundamental structure of a signed measure by asserting the existence of a partition of the space into two disjoint sets: one positive and one negative. This decomposition highlights the duality within a signed measure and provides a powerful tool for analyzing its properties. In our context, we will be applying the theory of Hahn decomposition to our *degree of uniformity* measure $m_{D}\left(P\right)$, which is a signed measure on a given probability space. As we will see later, we are able to apply the Hahn decomposition in a nested fashion in order to *quantify* and *locate* the concentration of inequality in different regions of a given probability space.

Next, we briefly define some key concepts to provide a clear foundation for introducing the Hahn decomposition.

**Definition** **1** **(Signed Measure).** *A signed measure μ on a measurable space (X,F) is a function μ:F→R such that:*

1. *μ(∅)=0 (the measure of the empty set is zero),*
2. *μ is countably additive, meaning for any sequence of pairwise disjoint sets ${\left\{A_{i}\right\}}_{i=1}^{\infty }\subseteq F$, we have:*
  $$\mu \left(\overset{\infty }{\underset{i=1}{⋃}}A_{i}\right)=\sum_{i=1}^{\infty }\mu \left(A_{i}\right),$$
  *where the series on the right may converge to a finite number or diverge to ±∞.*

**Definition** **2** **(Positive (negative, null) Set).** *A set A is called a positive (negative, null) set with respect to a signed measure μ if for every measurable subset E⊆A, we have:*

μ(E)≥0(≤0,=0).

We note that in the Definition 2 above, the word positive can be replaced with *strictly positive* when μ(E)>0, and negative can be replaced with *strictly negative* when μ(E)<0. We emphasize the difference between a positive set and a set that has a positive value for the signed measure. A positive set necessarily has to satisfy the property that all measurable subsets should also have positive or zero measure. However, the total measure of a set can be positive due to cancellation of positive and negative measure values of its disjoint parts, for example. Analogous statements are true for negative and null sets which are, respectively, different than sets that have a measure value that is negative or zero.

**Definition** **3** **(Hahn Decomposition).** *Given a signed measure μ defined on a measurable space (X,F), a partition of X into two disjoint sets P and N such that:*

1. *P is a positive set, i.e., μ(E)≥0 for all measurable subsets E⊆P,*
2. *N is a negative set, i.e., μ(E)≤0 for all measurable subsets E⊆N,*

*is defined as the Hahn decomposition (P,N) of the space X with respect to the signed measure μ.*

We state a theorem from [11] below that guarantees the existence of the Hahn decomposition for a given signed measure on a measure space.

**Theorem** **1.** *Given a signed measure μ on a measurable space (X,F), there exists a Hahn decomposition which is unique up to null sets.*

Theorem 1 means that given a Hahn decomposition $\left(P_{1},N_{1}\right)$, we could take a null set from $P_{1}$ and transfer it to $N_{1}$ (or vice-versa) to obtain another decomposition $\left(P_{2},N_{2}\right)$. Hence, any two decompositions differ only by null sets. However, as we will see later while applying this theorem to the degree of uniformity measure $m_{D}$ on a given probability space, the null set can be explicitly identified. This allows us to partition the original probability space into a disjoint triple (P,N,Z), where *P* is a strictly positive set, *Z* is a null set, and *N* is a strictly negative set. This removes the ambiguity related to the null set that is presented in Theorem 1.

In the next section, we introduce some background on the topic of mathematical diversity and the newly discovered *degree of uniformity* measure.

### 2.2. Shannon Diversity and Hill Numbers

Mathematical diversity (denoted as DK1 or ^1^*D* or just *D*) quantifies both the variety of categories within a distribution (richness) and how uniformly they are represented (evenness), as described in [7,8,9,10]. A probability distribution that is uneven can be converted into a Shannon Equivalent Equiprobable (SEE) distribution, which is uniform but reflects the same amount of informational uncertainty. For a discrete distribution where all categories have equal probabilities, diversity corresponds to the total count of categories. In continuous distributions, it is determined by the length of the interval on which the distribution is uniform, represented by the Lebesgue measure of the support. Any lack of uniformity reduces the computed diversity. For further details on mathematical diversity, refer to [7,12,13,14,15].

NOTE: We will summarize the key definitions and theorems on mathematical diversity from [1,2] that are pertinent to this study. In this paper, the subscript *K* is used to denote a discrete distribution, $\left\{k_{1},k_{2}\right\}$ represents a set of contiguous indices in a discrete subset, and (a,b) indicates continuous distributions or intervals representing parts of these distributions. When a result applies to both discrete and continuous cases, we will specify this explicitly and omit subscripts. The notation *I* will be used for the full support of a distribution: I={1,…,K} for discrete distributions and I=(a,b) for continuous distributions, as applicable.

**Definition** **4.** *(Shannon Diversity corresponding to q=1 for Hill numbers) Consider a discrete random variable X with support I={1,…K} (with K=∞ allowed) and its probabilities $p_{i}$, or a continuous random variable X with support I=(a,b) (with a=−∞ and b=+∞ allowed) and its probability density p(x). The diversity of the entire distribution* ^1^*D is defined as the length of the support of an equivalent uniform distribution that yields the same value of Shannon entropy H.*

Shannon entropy for discrete and continuous distributions is defined as below:

(1) $$H_{I}=-\sum_{i=1}^{K}p_{i}\ln\left(p_{i}\right);\;H_{I}=-\int_{\left(a,b\right)}p\left(x\right)\ln\left(p\left(x\right)\right)dx.$$

To avoid mathematical pathologies, we only consider probability distributions for which the entropy $H_{I}$ is finite. It has been shown [7,8,9,10] that Definition 4 implies that the total diversity ^1^*D* (for both continuous and discrete distributions) is given by:

(2) DI1=eHI.

We will focus exclusively on the case q=1 for the Hill numbers and, hence, will omit the left superscript 1, referring to the diversity simply as *D*. The choice of q=1 is made because it assigns equal weight to both richness and evenness.

For any subset P⊆{1,…,n}, we have already defined the partwise entropy $H_{P}$ and diversity $D_{P}=\exp\left(H_{P}\right)$. Let $c_{P}:=\sum_{i\in P}p_{i}$ denote the partwise mass. While we do not introduce a separate symbol, the ratio $D_{P}/c_{P}$ serves as a useful interpretive quantity; it reflects the effective diversity per unit mass within *P*, and will play a role in the analysis of uniformity and concentration.

We have spent quite a bit of time constructing and justifying the name *degree of uniformity* or *degree of inequality* for the ratio $\frac{D_{P}}{c_{P}}$ in [1,2,3]. We have also discussed the scale-free and self-contained nature of the ratio $\frac{D_{P}}{c_{P}}$ in [4]. We recall a few of the ideas from our previous papers here for the sake of continuity.

**Theorem** **2.** *Let $p_{i}$ with i∈I={1,…K} be a discrete probability distribution, with K=+∞ permitted. Let p(x) be a probability density function (pdf) on I=(a,b), with a=−∞ and b=+∞ permitted. Let $P=\underset{i}{⋃}P_{i}$ be a disjoint partition of a part P⊆I. Then the following is true for both discrete and continuous distributions:*

(3) $${\left(\frac{D_{P}}{c_{P}}\right)}^{c_{P}}=\prod_{P_{i}\in P}{\left(\frac{D_{P_{i}}}{c_{P_{i}}}\right)}^{c_{P_{i}}}.$$

We next define the degree of uniformity of a part $P=\{k_{1},k_{2}\}$ or $P=(x_{1},x_{2})$.

**Definition** **5.** *Let P denote a measurable subset of the form $\left\{k_{1},k_{2}\right\}$ for a discrete probability distribution or $\left(x_{1},x_{2}\right)$ for a continuous distribution. The ratio $\frac{D_{P}}{c_{P}}$ is termed as degree of uniformity or degree of inequality of the part P.*

We define the *degree of uniformity* measure [4] as follows:

**Definition** **6.** *Let $p_{i}$ with i∈I={1,…K} be a discrete probability distribution, with K=+∞ permitted. Alternatively, let p(x) be a probability density function (pdf) on I=(a,b), with a=−∞ and b=+∞ permitted. Let P be a general measurable subset (not necessarily contiguous) of I. We define a new signed measure on I called the degree of uniformity or degree of inequality measure for such a measurable subset P⊆I (irrespective of whether it is from a discrete or continuous distribution) by the following:*

(4) $$\;\left(\mathrm{Disc}.\right)\;m_{D}\left(P\right)=-\sum_{i\in P}p_{i}\ln\left(p_{i}D\right),$$

(5) $$\;\left(\mathrm{Cont}.\right)\;m_{D}\left(P\right)=-\int_{P}p\left(x\right)\ln\left(p\left(x\right)D\right).$$

**Remark** **1.** *The degree of uniformity measure $m_{D}$ in Definition 6 is with respect to the entire probability space I, where we have replaced the diversity $D_{I}$ with simply the letter D. This measure can also be defined for restrictions on proper subsets P⊊I with D replaced by $\frac{D_{P}}{c_{P}}$ in Definition 6. The degree of uniformity measure for the restriction to the set P will then be denoted by $m_{\frac{D_{P}}{c_{P}}}$. For the entire probability space I, we have that $\frac{D_{I}}{c_{I}}=D$ since $D_{I}=D$ and $c_{I}=1$.*

The degree of uniformity measure $m_{D}$ is a signed measure as seen in Definition 6. Given a measurable subset *P*, the sign of $m_{D}\left(P\right)$ indicates whether the given measurable subset *P* has a degree of uniformity that is less than, equal to, or greater than *D*, which is the degree of uniformity of the entire distribution. If we replace the *D* in $m_{D}\left(P_{2}\right)$ with the ratio $\frac{D_{P_{1}}}{c_{P_{1}}}$, then $m_{\frac{D_{P_{1}}}{c_{P_{1}}}}\left(P_{2}\right)$ can be used to compare the degree of uniformity of the parts $P_{1}$ and $P_{2}$ which can actually be parts from two different distributions. We state a theorem below that shows this fact in addition to other properties of $m_{D}\left(P\right)$ which were proved in [4].

**Theorem** **3.** *Let $p_{i}$ with $i\in I_{1}=\left\{1,\ldots K_{1}\right\}$ and $q_{i}$ with $i\in I_{2}=\left\{1,\ldots ,K_{2}\right\}$ be two different discrete probability distributions corresponding to random variables $X_{1}$ and $X_{2}$, respectively, with $K_{1}=+\infty$ and $K_{2}=+\infty$ permitted. Similarly let p(x) and q(x) be two different continuous probability distributions on $I_{1}=\left(a,b\right)$ and $I_{2}=\left(c,d\right)$ corresponding to random variables $X_{1}$ and $X_{2}$ respectively, with a,c=−∞ and b,d=+∞ allowed. Let $P_{1}$ and $P_{2}$ be measurable subsets from the probability spaces of $X_{1}$ and $X_{2}$ respectively. Then the following is true:*

(6) $$m_{\frac{D_{P_{1}}}{c_{P_{1}}}}\left(P_{2}\right)=c_{P_{2}}\ln\left(\frac{\left[\frac{D_{P_{2}}}{c_{P_{2}}}\right]}{\left[\frac{D_{P_{1}}}{c_{P_{1}}}\right]}\right),$$
(7) $$\frac{D_{P_{2}}}{c_{P_{2}}}=\left(\frac{D_{P_{1}}}{c_{P_{1}}}\right)\exp\left(\frac{m_{\frac{D_{P_{1}}}{c_{P_{1}}}}\left(P_{2}\right)}{c_{P_{2}}}\right),$$
(8) $$m_{\frac{D_{P_{1}}}{c_{P_{1}}}}\left(P_{2}\right)\;\left(\begin{array}{c} >\\ =\\ < \end{array}\right)\;0\;\Leftrightarrow \;\frac{D_{P_{2}}}{c_{P_{2}}}\;\left(\begin{array}{c} >\\ =\\ < \end{array}\right)\;\frac{D_{P_{1}}}{c_{P_{1}}},$$
(9) $$\frac{m_{\frac{D_{P_{1}}}{c_{P_{1}}}}\left(P_{2}\right)}{c_{P_{2}}}+\frac{m_{\frac{D_{P_{2}}}{c_{P_{2}}}}\left(P_{1}\right)}{c_{P_{1}}}=0.$$

This completes our brief introduction to the background material on mathematical diversity that is useful for this paper. In the next section, we try to connect the idea of Hahn decomposition to the degree of uniformity measure.

## 3. Hahn Decomposition of the Degree of Uniformity Measure $m_{D}$

The most important part of Theorem 3 is Equation (8). It says that if the measure $m_{\frac{D_{P_{1}}}{c_{P_{1}}}}$ has a positive (zero or negative) value for a given set $P_{2}$ then that means the degree of uniformity of $P_{2}$ is larger (equal to or smaller) than the degree of uniformity of $P_{1}$. This fact was explored in [4]. In this paper, however, we are motivated to take advantage of the Hahn decomposition as stated in Theorem 1, which guarantees the division of the probability space *I* into positive and negative sets *P* and *N* using the degree of uniformity measure $m_{D}\left(I\right)$, i.e., I=P∪N. The key observation here is that $m_{D}\left(P\right)\geq 0$ and $m_{D}\left(N\right)\leq 0$. Although Theorem 1 guarantees that the decomposition is unique up to null sets, in fact, as we will see later, we can explicitly obtain a description of the null set *Z* which satisfies $m_{D}\left(Z\right)=0$.

Hence, in our specific case of application of the Hahn decomposition theorem to the signed measure $m_{D}$ on the probability space *I*, we actually obtain a disjoint triplet I=P∪Z∪N. The set *P* in our case is called *strictly positive* and *N* is called *strictly negative*, and the *null* sets have been removed and placed into the set *Z*. In addition, for all measurable subsets A⊆P, B⊆Z, and C⊆N, we have that $m_{D}\left(A\right)>0$, $m_{D}\left(B\right)=0$, and $m_{D}\left(C\right)<0$. Hence the decomposition preserves the monotonicity with respect to the degree of uniformity measure $m_{D}$ in the following sense. The original probability space is split into three subsets: *P* has a larger degree of uniformity than *I*, *Z* has the same degree of uniformity as *I*, and *N* has a lesser degree of uniformity than *I*. Hence $\frac{D_{P}}{c_{P}}>\frac{D_{Z}}{c_{Z}}=D_{I}>\frac{D_{N}}{c_{N}}$. Furthermore for subsets A⊆P,B⊆Z,C⊆N, we have that $\frac{D_{A}}{c_{A}}>\frac{D_{B}}{c_{B}}=D_{I}>\frac{D_{C}}{c_{C}}$, i.e., all measurable subsets of P,Z, and *N* also follow the same monotonicity of order with respect to degree of uniformity. The monotonicity of ordering is preserved because of the fact that all measurable subsets of positive sets with respect to a given signed measure are necessarily positive as well, by definition. In what follows, we outline the general idea of the nested Hahn decomposition that we will construct in the rest of the paper. This will set the stage for the main results in this paper.

**Figure 1:**
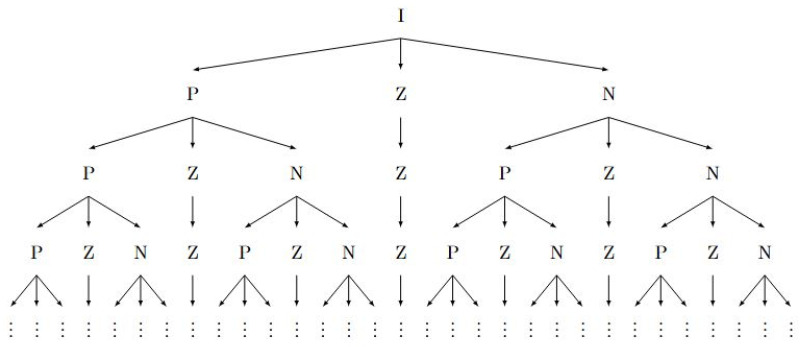
A general tree diagram depicting the nested Hahn decomposition of a given probability space *I*.

We can use Equation (8) in a nested fashion in the following way:

1. First, we start with the probability space *I* that is given to us, for which we have $c_{I}=1$ and $D_{I}=D$, the diversity of the entire distribution. We use the Hahn decomposition method for signed measures to the measure $m_{D}$ on the entire probability space *I* and split it into a strictly positive and strictly negative set *P* and *N*, respectively. As we will see during the construction, the null set can also be explicitly calculated and we will denote that by *Z*.
2. The strictly positive set *P* has a larger degree of uniformity, the null set *Z* has an equal degree of uniformity, and the negative set has a lesser degree of uniformity than the entire distribution, respectively. This is the first step in the decomposition.
3. Since the strictly positive set *P* has a degree of uniformity equal to $\frac{D_{P}}{c_{P}}$, we can use the signed measure denoted by $m_{\frac{D_{P}}{c_{P}}}$ to further subdivide *P* into three parts using the Hahn decomposition for $m_{\frac{D_{P}}{c_{P}}}$. The three parts will have a larger, equal, and lesser degree of uniformity, respectively, than *P*. Note that the measure $m_{D}$ is different than the measure $m_{\frac{D_{P}}{c_{P}}}$. The former splits the entire distribution into three parts and the latter splits just the positive set *P* into three parts.
4. The set *Z* obtained from step 1 cannot be split any further because it is uniform, and every measurable subset of a uniform distribution has the same degree of uniformity as the entire distribution. However, due to the preservation of monotonicity, *Z* is carried over into the next level at the same position as before.
5. The set *N* obtained in step 1 can be split in a similar way to the set *P* in step 3, as a result of the Hahn decomposition of the measure $m_{\frac{D_{N}}{c_{N}}}$ leading to three subsets *P*, *Z*, and *N*.
6. Hence, in the second level, we would in general have 7 partitions, the (P,Z,N) that came from *P*, the *Z* from the previous level that cannot be split any further, and the (P,Z,N) that came from *N*.
7. This procedure can be continued with the caveat that all the *Z*s obtained in previous levels get carried over into the next level and all the *P*s and *N*s get split into (P,Z,N). We use arrows to indicate parent and children in the form of a tree diagram as shown in Figure 1.

We next state and prove an important result relating to strictly positive, strictly negative, and null sets with respect to the degree of uniformity measure $m_{D}$. We present the theorems for discrete and continuous cases separately for clarity of reading:

**Theorem** **4.** *Let $p_{i}$ with i∈I={1,…K} be a discrete probability distribution, with K=+∞ permitted. Alternatively, let p(x) be a probability mass function (pmf) on I=(a,b), with a=−∞ and b=+∞ permitted. Let P be a general measurable subset (not necessarily contiguous) of I, and let $m_{D}$ denote the degree of uniformity measure on I. Then we have the following:*

(10) $$\begin{array}{ccc} \; & \; & P\;\mathrm{is}\;a\;\mathrm{strictly}\;\mathrm{positive}\;(\mathrm{strictly}\;\mathrm{negative},\;\mathrm{null})\;\mathrm{set}\;\mathrm{with}\;\mathrm{respect}\;\mathrm{to}\;m_{D}\\ & \; & \Leftrightarrow p_{i}<\left(>,=\right)\frac{1}{D}\;\forall i\in P \end{array}$$

**Proof.**  We will prove the theorem for the strictly positive property. The proof for strictly negative and null properties follow the same lines.

1. ⇐ We recall that $m_{D}\left(P\right)=-\sum_{i\in P}p_{i}\ln\left(p_{i}D\right)$ for the discrete case. Assume that $p_{i}<\frac{1}{D}\;\forall i\in P$. Let A⊆P be an arbitrary measurable subset of *P*. Then we have the following:
  $$p_{i}<\frac{1}{D}\;\forall i\in A\Rightarrow m_{D}\left(A\right)=-\sum_{i\in A}p_{i}\ln\left(p_{i}D\right)>0.$$
  This proves the ⇐ direction for the discrete case.
2. ⇒ Let *P* be a strictly positive subset of *I*. This means that every measurable subset *A* of *P* is also strictly positive. Let us assume that $p_{i}>\frac{1}{D}\;\forall i\in A$ for some measurable subset A⊆P. Then we have the following:
  $$m_{D}\left(A\right)=-\sum_{i\in A}p_{i}\ln\left(p_{i}D\right)<0,$$
  and this contradicts our assumption that *P* is strictly positive with respect to $m_{D}$, since we cannot have a measurable subset for which $m_{D}\left(A\right)<0$. This proves the ⇒ direction for the discrete case.

□

**Theorem** **5.** *Let $p_{i}$ with i∈I={1,…K} be a discrete probability distribution, with K=+∞ permitted. Alternatively, let p(x) be a probability density function (pdf) on I=(a,b), with a=−∞ and b=+∞ permitted. Let P be a general measurable subset (not necessarily contiguous) of I, and let $m_{D}$ denote the degree of uniformity measure on I. Then we have the following:*

(11) $$\begin{array}{ccc} \; & \; & \;\left(\mathrm{Cont}.\right)\;P\;\mathrm{is}\;a\;\mathrm{strictly}\;\mathrm{positive}\;(\mathrm{strictly}\;\mathrm{negative},\;\mathrm{null})\;\mathrm{set}\;\mathrm{with}\;\mathrm{respect}\;\mathrm{to}\;m_{D}\\ & \; & \Leftrightarrow p\left(x\right)<\left(>,=\right)\frac{1}{D}\;\forall x\in P. \end{array}$$

**Proof.** 

1. ⇐ We recall that $m_{D}\left(P\right)=-\int_{P}p\left(x\right)\ln\left(p\left(x\right)D\right)dx$ for the continuous case. Assume that $p\left(x\right)>\frac{1}{D}\;\forall x\in P$. Let A⊆P be an arbitrary measurable subset of *P*. Then we have the following:
  $$p\left(x\right)<\frac{1}{D}\;\forall x\in A\Rightarrow m_{D}\left(A\right)=-\int_{A}p\left(x\right)\ln\left(p\left(x\right)D\right)dx>0.$$
  This proves the ⇐ direction for the continuous case.
2. ⇒ Let *P* be a strictly positive subset of *I*. This means that every measurable subset *A* of *P* is also strictly positive. Let us assume that $p\left(x\right)>\frac{1}{D}\forall x\in A$ for some measurable subset A⊆P. Then we have the following:
  $$m_{D}\left(A\right)=-\int_{A}p\left(x\right)\ln\left(p\left(x\right)D\right)dx<0,$$
  and this contradicts our assumption that *P* is strictly positive with respect to $m_{D}$, since we cannot have a measurable subset for which $m_{D}\left(A\right)<0$. This proves the ⇒ direction for the continuous case.

□

We have the following immediate corollary:

**Corollary** **1.** *Let $p_{i}$ with i∈I={1,…K} be a discrete probability distribution, with K=+∞ permitted. Alternatively, let p(x) be a probability density function (pdf) on I=(a,b), with a=−∞ and b=+∞ permitted. Let A and P with A⊂P be general measurable subsets (not necessarily contiguous) of I, and let $m_{\frac{D_{P}}{c_{P}}}$ denote the degree of uniformity measure on the restriction P⊂I. Then we have the following:*

(12) $$\begin{array}{ccc} \; & \; & \;\left(\mathrm{Disc}.\right)\;A\;\mathrm{is}\;a\;\mathrm{strictly}\;\mathrm{positive}\;(\mathrm{strictly}\;\mathrm{negative},\;\mathrm{null})\;\mathrm{set}\;\mathrm{with}\;\mathrm{respect}\;\mathrm{to}\;m_{\frac{D_{P}}{c_{P}}}\\ & \; & \Leftrightarrow p_{i}<\left(>,=\right)\frac{c_{P}}{D_{P}}\;\forall i\in A \end{array}$$

(13) $$\begin{array}{ccc} \; & \; & \;\left(\mathrm{Cont}.\right)\;A\;\mathrm{is}\;a\;\mathrm{strictly}\;\mathrm{positive}\;(\mathrm{strictly}\;\mathrm{negative},\;\mathrm{null})\;\mathrm{set}\;\mathrm{with}\;\mathrm{respect}\;\mathrm{to}\;m_{\frac{D_{P}}{c_{P}}}\\ & \; & \Leftrightarrow p\left(x\right)<\left(>,=\right)\frac{c_{P}}{D_{P}}\;\forall x\in A. \end{array}$$

**Proof.**  The proof follows the same lines as Theorems 4 and 5 by applying those steps to the measure $m_{\frac{D_{P}}{c_{P}}}$. □

The main points of Corollary 1 are the following:

1. We first note that for P=I, we have that the total probability is 1, i.e., $c_{I}=1$ and the diversity is $D_{I}$ (which we have written as *D* in this paper). Hence we recover Theorems 4 and 5.
2. If P⊊I then the degree of uniformity measure restricted to *P* is $m_{\frac{D_{P}}{c_{P}}}$ as seen in Remark 1. Hence, we can now obtain the Hahn decomposition of the proper subset *P* with respect to $m_{\frac{D_{P}}{c_{P}}}$, leading to three subsets that are more uniformly, equally uniformly, or less uniformly distributed, respectively, than *P*.
3. From point number 2, it is clear that the subsets obtained after decomposing *P* can further be decomposed using the degree of uniformity measure restricted to those subsets and, hence, this decomposition process is nested and can be continued ad infinitum until there are no new sets that are created. This is the main idea of the nested Hahn decomposition in this paper.

Next, we state and prove a theorem that explicitly calculates the Hahn decomposition of the probability space *I* or any of its subsets. We state and prove the theorem for an arbitrary measurable subset A⊂I. This means that the starting point of the decomposition can be any measurable subset of *I* and not just *I* itself. We will revisit this idea later.

**Theorem** **6.** *Let $p_{i}$ with i∈I={1,…K} be a discrete probability distribution, with K=+∞ permitted. Alternatively, let p(x) be a probability density function (pdf) on I=(a,b), with a=−∞ and b=+∞ permitted. Let A⊆I be an arbitrary measurable subset and $m_{\frac{D_{A}}{c_{A}}}$ be the degree of uniformity measure restricted to A. Then, A has the following disjoint Hahn decomposition triplet $\left(P_{A},Z_{A},N_{A}\right)$ with respect to the measure $m_{\frac{D_{A}}{c_{A}}}$ where $P_{A}$ is a strictly positive set, $Z_{A}$ is a null set, and $N_{A}$ is a strictly negative set:*

(14) $$\begin{array}{cc} & A=P_{A}\cup Z_{A}\cup N_{A}, \end{array}$$

(15) $$\begin{array}{cc} & \;\left(\mathrm{Disc}.\right)\;P_{A}=\left\{i\in A:p_{i}<\frac{c_{A}}{D_{A}}\right\}\;\mathrm{or}\;\left(\mathrm{Cont}.\right)\;P_{A}=\left\{x\in A:p\left(x\right)<\frac{c_{A}}{D_{A}}\right\} \end{array}$$

(16) $$\begin{array}{cc} & \;\left(\mathrm{Disc}.\right)\;Z_{A}=\left\{i\in A:p_{i}=\frac{c_{A}}{D_{A}}\right\}\;\mathrm{or}\;\left(\mathrm{Cont}.\right)\;Z_{A}=\left\{x\in A:p\left(x\right)=\frac{c_{A}}{D_{A}}\right\} \end{array}$$

(17) $$\begin{array}{cc} & \;\left(\mathrm{Disc}.\right)\;N_{A}=\left\{i\in A:p_{i}>\frac{c_{A}}{D_{A}}\right\}\;\mathrm{or}\;\left(\mathrm{Cont}.\right)\;N_{A}=\left\{x\in A:p\left(x\right)>\frac{c_{A}}{D_{A}}\right\} \end{array}$$

*Furthermore, $P_{A},Z_{A}$, and $N_{A}$ are the largest strictly positive, null, and strictly negative subsets of A with respect to the signed measure $m_{\frac{D_{A}}{c_{A}}}$, respectively.*

**Proof.**  We first note that the same proof works for A=I as well. Given that A⊆I in general, we can consider a renormalization of the probabilities to $\frac{p_{i}}{c_{A}}$ in the discrete case and the density to $\frac{p(x)}{c_{A}}$ in the continuous case. With the renormalization, *A* is now a bona fide probability space in its own right. Let us denote the sigma-algebra of all events in the sample space *A* by P. An application of Theorem 1 to the signed measure denoted by $m_{\frac{D_{A}}{c_{A}}}$ on the measure space (A,P) splits the set *A* into $A=P_{A}\cup N_{A}$, where $P_{A}$ is a positive set and $N_{A}$ is a negative set. The decomposition is unique up to null sets. However, we note that from Corollary 1, the null set part of *A* with respect to the signed measure $m_{\frac{D_{A}}{c_{A}}}$ (denoted by $Z_{A}$) is given by the following:

$$Z_{A}=\left\{i\in A:p_{i}=\frac{c_{A}}{D_{A}}\right\}\;\mathrm{or}\;Z_{A}=\left\{x\in A:p\left(x\right)=\frac{c_{A}}{D_{A}}\right\},$$

for the discrete and continuous cases, respectively. Hence, the ambiguity of where to put the null set can be resolved by simply removing $Z_{A}$ and treating it as a third part of the decomposition. After removing $Z_{A}$ (the null set portions from $P_{A}$ and $N_{A}$), $P_{A}$ will be strictly positive and $N_{A}$ will be strictly negative, where we use the same notation for $P_{A}$ and $N_{A}$ even after removing $Z_{A}$ from them. Equations (15) and (17) will then follow from Corollary 1. Hence, for both the discrete and continuous cases, we have a disjoint Hahn decomposition given by $I=P_{A}\cup Z_{A}\cup N_{A}$.

To show that $P_{A}$ is the largest strictly positive subset of *A* follows from Corollary 1 since all strictly positive subsets P⊂A should necessarily satisfy $p_{i}<\frac{c_{A}}{D_{A}}\;\forall i\in P$, or $p\left(x\right)<\frac{c_{A}}{D_{A}}\;\forall x\in P$, we can collect all such *i*s in the discrete case or *x*s in the continuous case and that will be the largest positive set. However, $P_{A}$ is precisely this set by Equation (15). To show that $Z_{A}$ is the largest null subset and $N_{A}$ is the largest strictly negative subset follows the same line of argument.

This proves the theorem. □

Theorem 6 allows us to decompose any subset A⊆I into a disjoint triplet $\left(P_{A},Z_{A},N_{A}\right)$ where $P_{A}$ is strictly positive, $Z_{A}$ is null and $N_{A}$ is strictly negative with respect to the measure $m_{\frac{D_{A}}{c_{A}}}$, which is the degree of uniformity measure for the set A⊆I. This lays down a concrete framework to recursively decompose $P_{A}$ and $N_{A}$ further into $\left(P_{P_{A}},Z_{P_{A}},N_{P_{A}}\right)$ and $\left(P_{N_{A}},Z_{N_{A}},N_{N_{A}}\right)$ with respect to the measures $m_{\frac{D_{P_{A}}}{c_{P_{A}}}}$ and $m_{\frac{D_{N_{A}}}{c_{N_{A}}}}$, respectively. A few things are of importance here:

Since $P_{A}$ is strictly positive with respect to $m_{\frac{D_{A}}{c_{A}}}$, it has a larger degree of uniformity than the set *A*. Furthermore, every subset of $P_{A}$ is also strictly positive with respect to $m_{\frac{D_{A}}{c_{A}}}$ and hence the sets from the next level of decomposition $\left(P_{P_{A}},Z_{P_{A}},N_{P_{A}}\right)$ are guaranteed to be strictly positive with respect to $m_{\frac{D_{A}}{c_{A}}}$ and hence are guaranteed to have a higher degree of uniformity than *A*. By the same token, $N_{A}$ has a lower degree of uniformity than *A* and so do its subsets $\left(P_{N_{A}},Z_{N_{A}},N_{N_{A}}\right)$. Hence, the degree of uniformity preserves the ordering within subsets.

The set $Z_{A}$ is a uniform distribution, and by definition every subset of $Z_{A}$ has the same degree of uniformity as $Z_{A}$ itself. Hence, it cannot be decomposed any further. However, since $\frac{D_{P_{A}}}{c_{P_{A}}}>\frac{D_{Z_{A}}}{c_{Z_{A}}}=\frac{D_{A}}{c_{A}}>\frac{D_{N_{A}}}{c_{N_{A}}}$, to preserve the ordering of degree of uniformity in the subsequent levels, $Z_{A}$ needs to be carried over into all subsequent levels maintaining its position in the ordering. This will preserve the decreasing order of degree of uniformity for sets in each level from left to right in Figure 1.

We reiterate that degree of uniformity is synonymous with degree of inequality in the sense that they are reciprocals of each other. The ratio $\frac{D_{P}}{c_{P}}$ tells us the amount of SEE equivalent extent of the random variable situated per unit cumulative frequency in *P* and is a direct indication of how uniform the random variable is in *P*. Hence, the tree diagram in Figure 1 is actually a decomposition of the original probability space *I* into subsets with increasing degree of inequality $\frac{c}{D}$ from left to right in a given horizontal level, and an increasing level of granularity as we go down the levels. This justifies the term *localization* of inequality since we can pinpoint the subsets in a given level where there is a larger localization of inequality. In fact, for each of the subsets in a given level, we can explicitly calculate the degree of inequality $\frac{c}{D}$ and compare it with that of other subsets in the same level. We can form ratios of degrees of inequality of subsets in a level to describe how much more or less unequally distributed two sets are compared to each other, or equivalently how much more or less concentrated the random variable is on the two sets.

## 4. Examples

We chose the exponential and binomial distributions as quick warm-up classical demonstrations of the nested Hahn decomposition method. The third example is a hypothetical distribution of disease contraction across age groups and the last one is an example of distribution of stress on a beam. These two examples are hypothetical and demonstrate the usefulness of our method for distributions obtained from real-life data. We end the examples section by showing a bona fide null set given that the previous examples did not admit a null set during the decomposition. For the disease contraction and the beam examples in this section, a standalone tree structure from [16] was used to do the nested Hahn decomposition in Matlab, whereas the exponential and the binomial examples were done by hand. We state the recursive step to compute the subdivisions of a general measurable subset *A* below:

1. Compute $\frac{c_{A}}{D_{A}}$.
2. Divide *A* into $P_{A}=\left\{i\in A:p_{i}<\frac{c_{A}}{D_{A}}\right\}$, $Z_{A}=\left\{i\in A:p_{i}=\frac{c_{A}}{D_{A}}\right\}$ and $N_{A}=\left\{i\in A:p_{i}>\frac{c_{A}}{D_{A}}\right\}$ in the discrete case or $P_{A}=\left\{x\in A:p\left(x\right)<\frac{c_{A}}{D_{A}}\right\}$, $Z_{A}=\left\{x\in A:p\left(x\right)=\frac{c_{A}}{D_{A}}\right\}$ and $N_{A}=\left\{x\in A:p\left(x\right)>\frac{c_{A}}{D_{A}}\right\}$ in the continuous case.
3. Repeat steps (1) and (2) with the set *A* replaced by $P_{A}$ and $N_{A}$ to compute the partitions in the next level. Carry $Z_{A}$ over to the next level at the same horizontal position as the current level.

### 4.1. Exponential Distribution with λ=2

**Figure 2:**
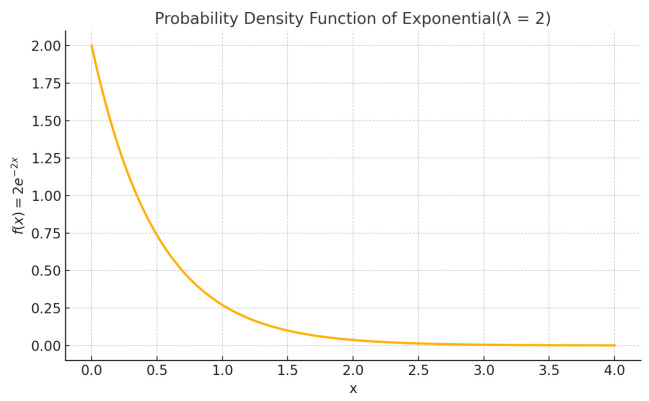
Probability density of the exponential distribution with λ=2.

**Figure 3:**
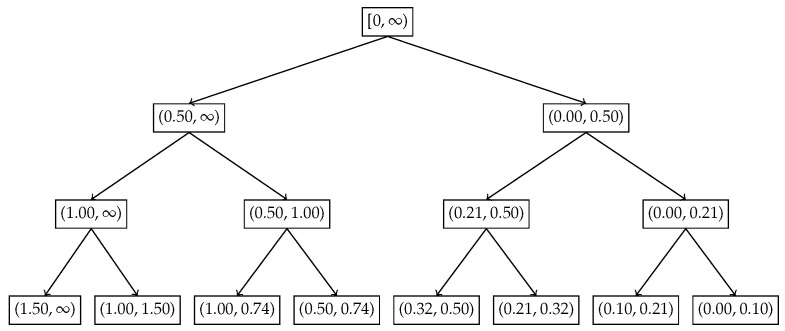
Exponential distribution with λ=2: Subsets of the Hahn decomposition.

**Figure 4:**
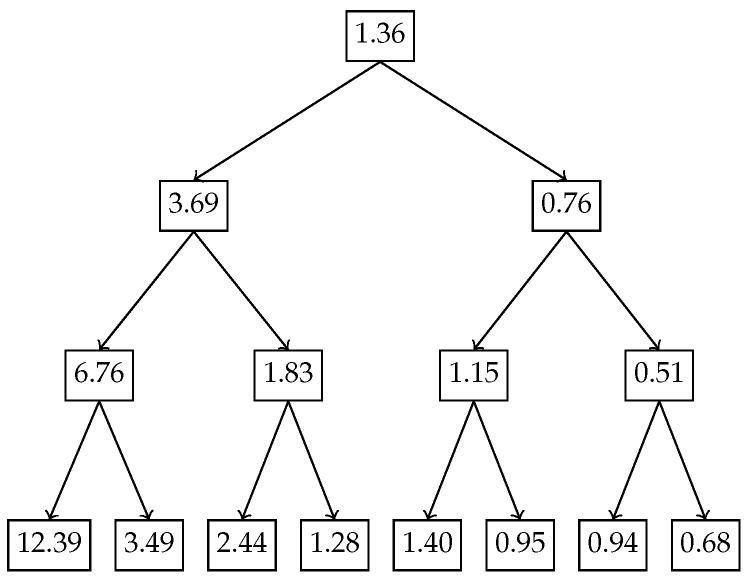
Exponential distribution with λ=2: Tree depicting the degree of uniformity of subsets from the nested Hahn decomposition.

In the first example, we apply the nested Hahn decomposition method to the exponential distribution with λ=2. We show the probability density function in Figure 2, the tree with the subsets in Figure 3 and the tree with the degrees of uniformity show in Figure 4, respectively. Intuitively, from Figure 2, it is clear that the distribution becomes more and more uniform as x→∞. Hence, we expect that the subsets to the right have a higher degree of uniformity as opposed to the ones closer to x=0.

Figure 3 and Figure 4 illustrate how the inequality is localized across recursive subdivisions of the exponential distribution using the recursive Hahn decomposition. The Hahn decomposition of the exponential distribution with rate parameter λ=2 exhibits a consistent monotonic ordering of the degree of uniformity, quantified by the ratio D/c, across all levels of refinement. At the second level, the probability space [0,∞) is split into four disjoint intervals: (1.00,∞), (0.50,1.00), (0.21,0.50), and (0.00,0.21), with corresponding D/c values of 6.76, 1.83, 1.15, and 0.51. These values decrease steadily from left to right, and the ratios between them are revealing: the uniformity in (1.00,∞) is approximately 3.7 times greater than in (0.50,1.00), over 5.8 times greater than in (0.21,0.50), and over 13 times greater than in (0.00,0.21), underscoring the decrease in degree of uniformity toward the origin. At the third level, each Level 2 part is further refined, producing eight sub-intervals: (1.50,∞), (1.00,1.50), (1.00,0.74), (0.50,0.74), (0.32,0.50), (0.21,0.32), (0.10,0.21), and (0.00,0.10), with D/c values of 12.39, 3.49, 2.44, 1.28, 1.40, 0.95, 0.94, and 0.68, respectively. Here too, the most uniform region, (1.50,∞), has a D/c value nearly 5 times that of (1.00,1.50) and over 18 times that of the least uniform region (0.00,0.10). These sharp contrasts quantitatively reinforce how the Hahn decomposition isolates areas of varying inequality, with the recursive partitioning honing in on regions of concentrated probability and diminished uniformity. Although the decomposition can be continued further, we chose to stop after 3 levels since there is no natural end to the process due to the continuous nature of the distribution.

### 4.2. Binomial Example B(8,0.4)

**Figure 5:**
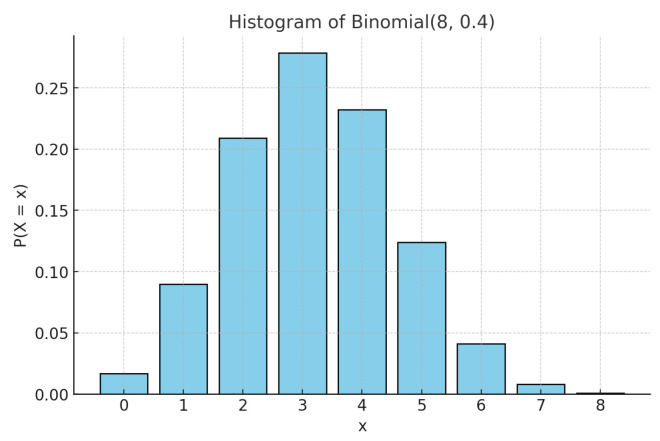
Histogram of the Binomial distribution B(8,0.4).

**Figure 6:**
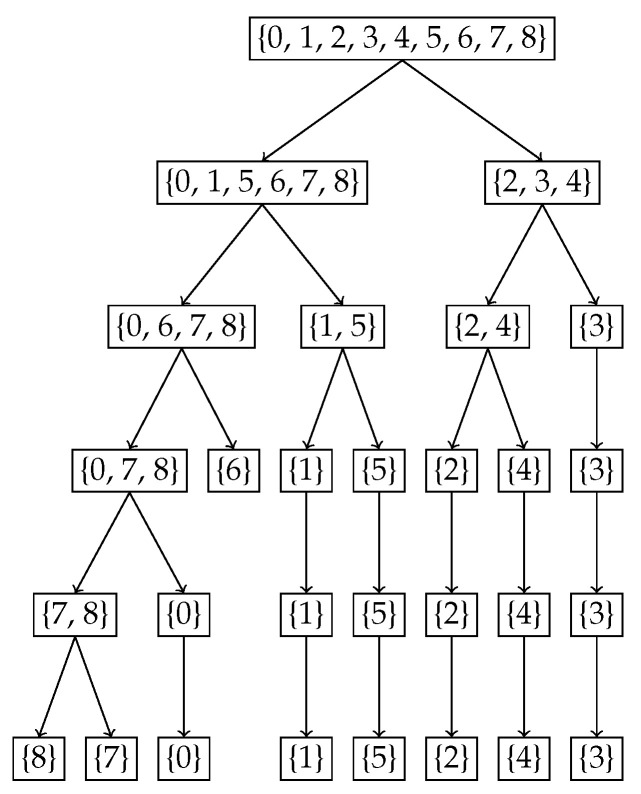
Binomial distribution B(8,0.4) tree depicting the subsets of the nested Hahn decomposition.

**Figure 7:**
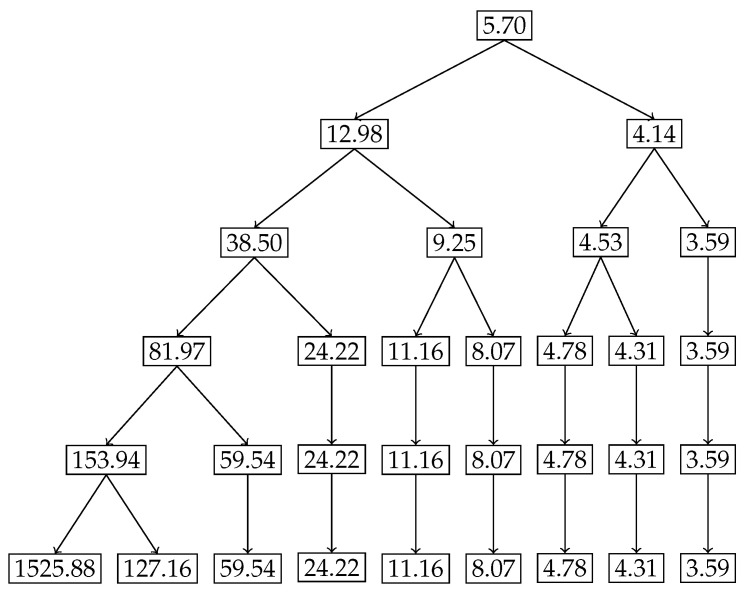
Binomial distribution B(8,0.4) tree depicting the degree of uniformity of subsets from the nested Hahn Decomposition.

For the second example, we chose the binomial distribution B(8,0.4). Given the discrete and finite nature of this distribution, we expect the nested Hahn decomposition to stop after a few levels. We show the histogram of the original distribution in Figure 5, the tree showing the subsets from the nested Hahn decomposition in Figure 6 and the tree showing the corresponding degrees of uniformity of subsets in Figure 7, respectively. Intuitively we expect the center portion of the distribution to be less uniformly distributed compared to the tails. This is precisely what we see in both trees. The decomposition stops eventually when all the subsets are singletons.

Figure 6 and Figure 7 demonstrate how the monotonicity of the degree of uniformity is preserved across recursive subdivisions for the Binomial distribution B(8,0.4). In the Hahn decomposition of the Binomial(8,0.4) distribution, the sample space {0,1,2,…,8} is partitioned at Level 2 into four disjoint subsets: {0,6,7,8}, {1,5}, {2,4}, and {3}, with corresponding D/c values of 81.97, 24.22, 8.07, and 3.59, respectively. These values decrease consistently from left to right, indicating increasing inequality and decreasing degree of uniformity. Comparing the ratios, the first part {0,6,7,8} has a D/c over 3.4 times greater than {1,5}, over 10 times greater than {2,4}, and over 22 times greater than {3}, which captures the point of maximum probability and hence degree of uniformity. At the next level of refinement, the subsets are further decomposed into {7,8}, {0}, {1}, {5}, {2}, {4}, and {3}, with associated D/c values of 153.94, 59.54, 24.22, 11.16, 8.07, 4.78, 3.59. The singleton {8} stands out with a strikingly high D/c value of 1525.88, reflecting maximal uniformity in an outcome with very low probability mass. This sharp contrast further illustrates how the recursive Hahn decomposition localizes regions of high and low uniformity: subsets with rare outcomes (e.g., {8} and {0}) exhibit high entropy per unit mass due to their dispersion in a skewed distribution, while concentrated mass points (e.g., {3}) correspond to minimal uniformity. These results echo the patterns seen in the exponential case, but within a discrete framework, reinforcing the mathematical and intuitive consistency of the approach.

### 4.3. Disease Distribution Across Age Groups

**Figure 8:**
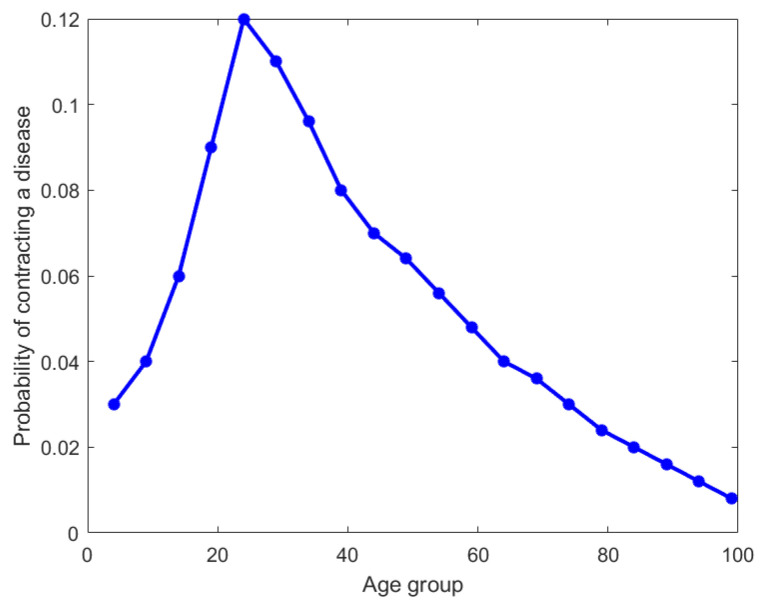
Original probability distribution of disease contraction across 20 age groups.

For the third example, we analyze a hypothetical dataset that is a distribution of incidences of a particular disease across 20 age groups. As seen in Figure 8 (the original distribution), the age groups are 0–4, 5–9, etc., until 95–99. For brevity, we have chosen to plot the upper bound of each interval on the x-axis. It is clear that this disease has a maximum incidence of 12% for the age group of 20–24 and tapers off on either side. We have applied our nested Hahn decomposition algorithm to quantify and locate the inequality of incidence in disease.

**Figure 9:**
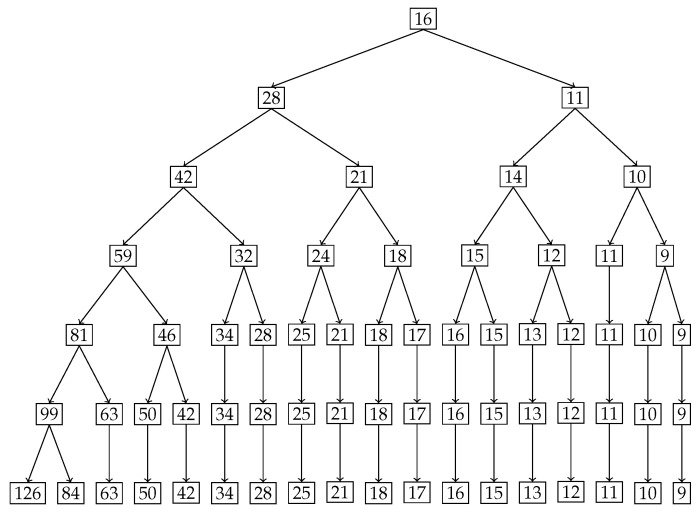
Tree diagram obtained from the nested Hahn decomposition of the disease contraction data where the degree of uniformity of the parts are shown in the form of numbers at each node.

**Figure 10:**
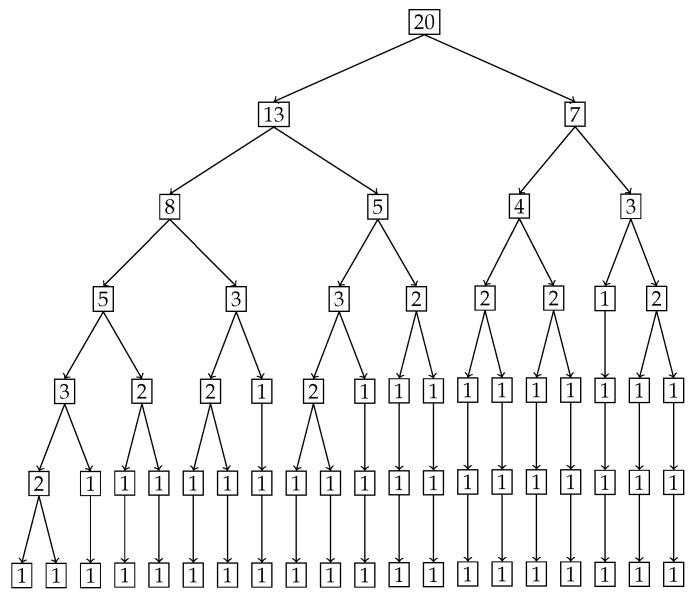
Tree diagram obtained from the nested Hahn decomposition of the disease contraction data where the number of elements of the parts are shown in the form of numbers at each node.

In Figure 9, the tree diagram obtained from the nested Hahn decomposition algorithm is shown with the degree of uniformity of parts shown as numbers. For example, the entire distribution has a degree of uniformity of 16 which after splitting in the first level gives two parts, one with a $\frac{D}{c}=28$ on the left and $\frac{D}{c}=11$ on the right. There are no *Z* (uniform) parts in this example. We see that there are 6 unique levels with the 7th level being a repeat, thereby serving as a stopping criterion for the algorithm. To keep our example simple, we only consider integer ages and assume that the ages are rounded down to the nearest integer. Hence an age of 3.5 years will be recorded as 3 years. We also chose to round up the degree of uniformity to the closest integer to avoid writing decimals in the tree diagram. Figure 9 shows the degree of uniformity of each part at each level. Figure 10 shows the number of elements in each part on the same tree diagram as Figure 9.

We explore the meaning of the tree diagram in Figure 9. The original dataset has 20 elements. We see that after level 4, there are way too many sub-parts with a single age group in them. We choose to discuss levels 2 and 3 to show how our method quantifies and locates the inequality of incidence of disease. We also note that after level 3, the right branch divides into sub-parts that all have more or less the same degree of uniformity. However, the left sub-part has a lot of inequality left as seen in the wide variation of degree of uniformity. We could discuss all the sub-parts in all levels, but in general, we choose levels with substantial or important age groups that are of interest from prior knowledge, or choose levels with decent sizes of sub-parts if needed.

**Figure 11:**
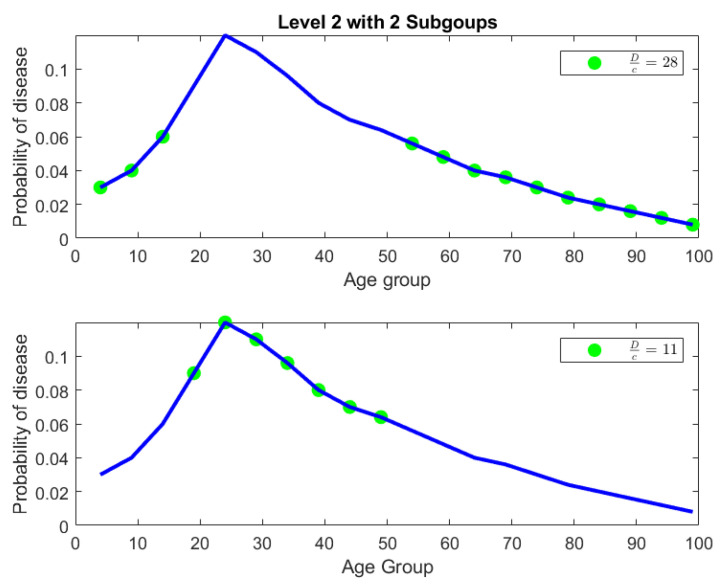
The parts in Level 2 of the disease contraction tree shown as green dots overlayed on the original distribution shown as a blue line.

**Figure 12:**
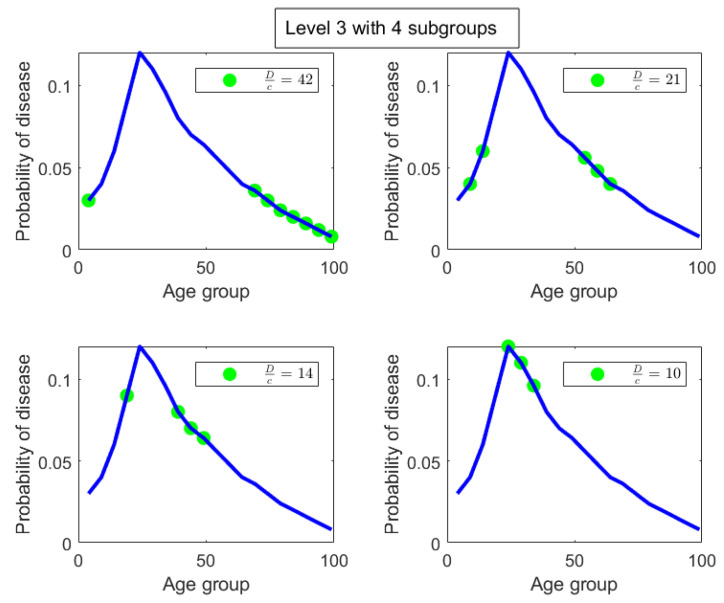
The parts in Level 4 of the disease contraction tree shown as green dots overlayed on the original distribution shown as a blue line.

1. **Level 2:** In level 2, we have two parts. The first part is ages $P_{1}=\left[0,14\right]\cup \left[50,99\right]$ with a degree of uniformity of 28. The second part is ages $P_{2}=\left[15,49\right]$ with a degree of uniformity of 11. Both parts in Level 2 are shown in Figure 11. There is a concentration of incidence of disease in the age group $P_{2}=\left[15,49\right]$ that is more than twice (in fact the ratio is $\frac{\frac{D_{P_{1}}}{c_{P_{1}}}}{\frac{D_{P_{2}}}{c_{P_{2}}}}=\frac{28}{11}$) the concentration of disease in the age group $P_{1}=\left[0,14\right]\cup \left[50,99\right]$. Alternatively speaking, the age group [0,14]∪[50,99] is $\frac{28}{11}$ times more uniformly distributed compared to the age group [15,49]. It is already clear at this point that the age group [15,49] contains the bulk of incidence of disease, entropically speaking. We also note that by construction of the Hahn decomposition, we are automatically guaranteed that all sub-parts of the age group [0,14]∪[50,99] will have a larger degree of uniformity than the entire distribution (the previous level). Similarly all sub-parts of [15,49] are guaranteed to automatically have a degree of uniformity smaller than the entire distribution (the previous level). In fact, it is clear from Figure 9 that monotonicity of degree of uniformity is preserved as we go down the levels, which was an important consideration as explained in the introduction.
2. **Level 3:** We note that in Level 3, the age group [0,14]∪[50,99] from Level 2 splits into 2 sub-parts, namely, [0,4]∪[65,99] and [5,14]∪[50,64]. Those have $\frac{D}{c}$ values of 42 and 21, respectively, and are shown in the top-left and top-right pictures in Figure 12 along with their degrees of uniformity labeled in the respective figures. Clearly we are further isolating the inequality within the [0,14]∪[50,99] age group from Level 2, as the new sub-part [5,14]∪[50,64] is twice as concentrated in disease incidence as the new sub-part [0,4]∪[65,99]. The other part from Level 2, namely the age group [15,49], splits into 2 sub-parts as well, given by [15,19]∪[35,49] and [20,34]. These two sub-parts are shown in the bottom-left and bottom-right pictures in Figure 12. Those have $\frac{D}{c}$ values of 14 and 10, respectively, which are shown in the labels of the pictures. Hence, these two new sub-parts have comparable concentration of disease incidence. However, the sub-part [20,34] has approximately twice the concentration of disease incidence as the subpart [5,14]∪[50,64] in the same level (Level 3).

We stop the analysis with these two levels but we make the following points:

1. We started with entire distribution of 20 age groups. However, we could have started with any sub-part of the distribution and the same algorithm can be used to construct a tree diagram.
2. It is clear from the analysis of the two levels (Level 2 and Level 3) that not only are we able to locate the inequality in the form of explicit age groups, but we are also able to quantify the relative concentrations of inequality between each sub-part.
3. In this example, there were no null sets that were formed since all the splits were binary. That is due to the monotonicity of the probability values. We do see in the last level (Level 6) that there are some sub-parts with two age groups that do not split. They are uniform distributions and they appear only at the end; however, they are not null sets as seen by their non-zero degree of uniformity values seen in Figure 9.
4. To reiterate, we have successfully maintained the monotonicity of degree of uniformity across subsets, i.e., to repeat, measurable subsets of two different measurable sets $S_{1}\subset P_{1}$ and $S_{2}\subset P_{2}$ with the ordering $\frac{D_{P_{1}}}{c_{P_{1}}}>\frac{D_{P_{2}}}{c_{P_{2}}}$ also follow the same ordering of degree of inequality, i.e., $\frac{D_{S_{1}}}{c_{S_{1}}}>\frac{D_{S_{2}}}{c_{S_{2}}}$.
5. We also reiterate that the ratio $\frac{D_{P}}{c_{P}}$ is the degree of uniformity, however its reciprocal $\frac{c_{P}}{D_{P}}$ is the degree of localization in the part *P*. For example, in Level 2, with $P_{1}=\left[0,14\right]\cup \left[50,99\right]$ and $P_{2}=\left[15,49\right]$, we have that $\frac{D_{P_{1}}}{c_{P_{1}}}=28$ and $\frac{D_{P_{2}}}{c_{P_{2}}}=11$ for the two sub-parts generated. This means that $P_{1}$ is $\frac{28}{11}$ times more uniformly distributed than $P_{2}$, or equivalently $P_{2}$ is $\frac{28}{11}$ times concentrated (or unequally distributed) than $P_{1}$. This means that the population sub-group in part $P_{2}$ is approximately 2.5 times more susceptible to the disease than the population sub-group in part $P_{1}$.
6. Level 2 tells us that the age group [15,49] is more than twice as vulnerable to this disease compared to the ages given by [0,14]∪[50,99].
7. Level 3 delves deeper into both these subgroups to further isolate the inequality. In particular, ages [5,14]∪[50,64] are twice as vulnerable as ages [0,4]∪[65,99]. Also, ages [20,34] are twice are vulnerable as [5,14]∪[50,64].
8. We can continue to dig deeper by analyzing further levels if the goal is to compare age groups with smaller intervals by repeating the same analytical ideas presented with Levels 2 and 3. The quantification of inequality through the idea of degree of uniformity are not visually clear just by looking at the sub-parts, especially given that these are unions of disjoint age groups and not a single contiguous age group. We have used the idea of entropy and diversity to systematically locate and quantify the inequality of incidence of disease in the original distribution.

We end the example by stating that the algorithm definitively identifies the vulnerable age groups within the original distribution and quantifies the degree of inequality (vulnerability) with respect to incidence of disease. This information can be invaluable for medical practitioners while making health care decisions for an individual patient (or groups of patients in the case of community care) based on the age (or age groups) present.

### 4.4. Stress Distribution Across a Beam

**Figure 13:**
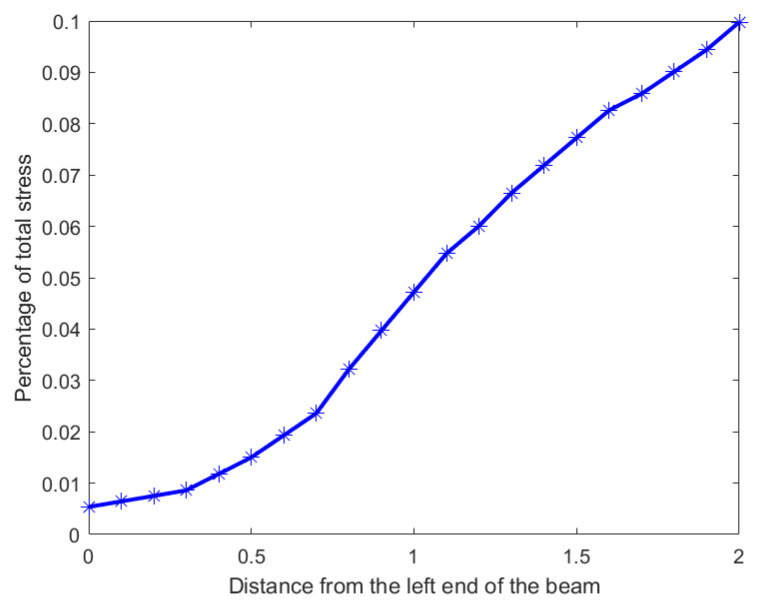
Percentage distribution of stress across a 2 m beam.

In our second example we use a hypothetical dataset depicting the stress distribution across a 2 m beam. Analyzing the inequality of distribution of stress across such a beam will allow engineers to predict and design beams with material compositions that can withstand the stress distribution. The original stress distribution is shown in Figure 13, the x-axis denotes the position on the beam from 0 m to 2 m in 0.1 m increments, and the y-axis shows the percentage of total stress at each position. It is clear from the distribution that the stress percentages increase monotonically from left to right with a maximum of 10 percent at the right end, namely at 2 m. To locate and quantify the inequality in distribution of stress across the beam, we have applied our nested Hahn decomposition to this distribution.

**Figure 14:**
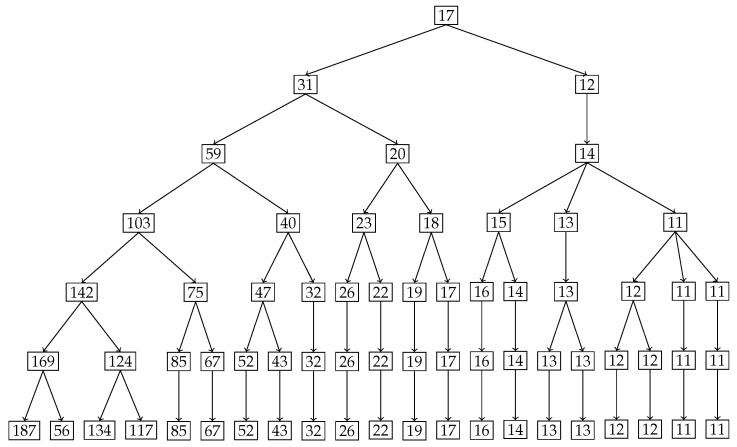
Tree diagram obtained from the nested Hahn decomposition of the stress on a beam data where the degree of uniformity of the parts are shown in the form of numbers at each node.

**Figure 15:**
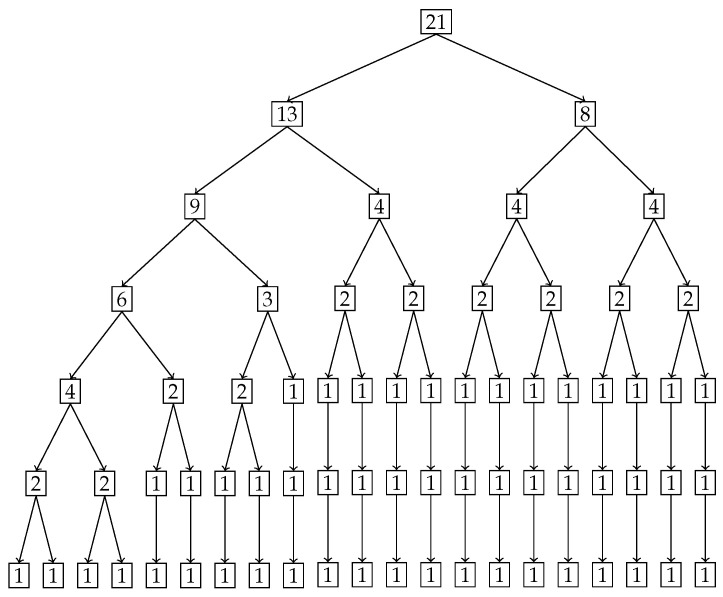
Tree diagram obtained from the nested Hahn decomposition of the stress on a beam data where the number of elements of the parts are shown in the form of numbers at each node.

In Figure 14, the degree of uniformity of parts is shown on the tree diagram obtained from the nested Hahn decomposition algorithm. It is clear from the tree diagram that there are 6 levels obtained from the decomposition, not including the entire distribution. We see that the degree of uniformity of the entire distribution is 17. In level 2, the entire beam is split into two parts, i.e., [0,1.2] and [1.3,2] meters. Looking at the subdivisions of the [1.3,2] meter part across further levels, almost every sub-part across further levels have the same degree of uniformity. However, the [0,1.2] meter part sub-divides into sub-parts across further levels with large variations in degree of uniformity. This indicates that the [0,1.2] meter part has a larger concentration of stress within it as seen in the segregation of values in degree of uniformity within its further sub-parts. Figure 15 shows the number of elements in each sub-part generated by the tree diagram. We note that after level 4, the right half of the tree diagram contains singletons and hence there are no more subdivision, whereas the left half has a few more levels of subdivisions until singletons appear there as well. Due to the monotonically increasing nature of the original distribution in Figure 13, there are no *Z* parts in the tree in this example as well, i.e., all splits are binary and not ternary.

**Figure 16:**
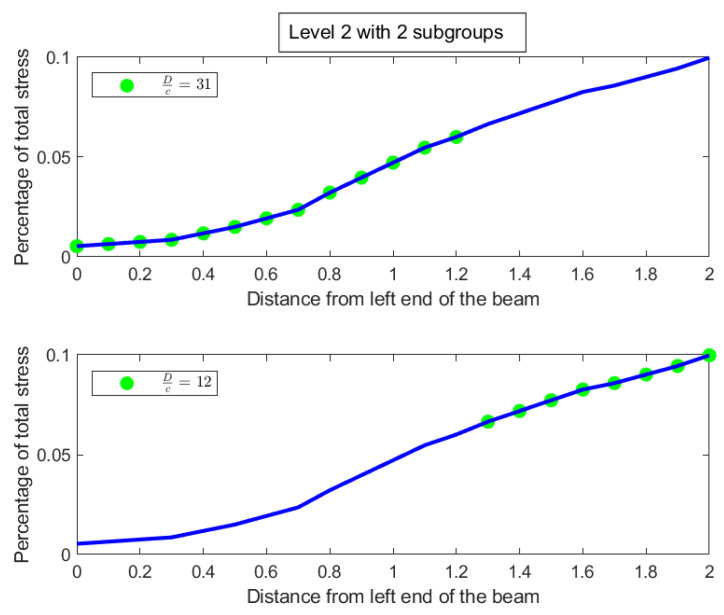
The parts in Level 2 of the stress on a beam tree shown as green dots overlayed on the original distribution shown as a blue line.

**Figure 17:**
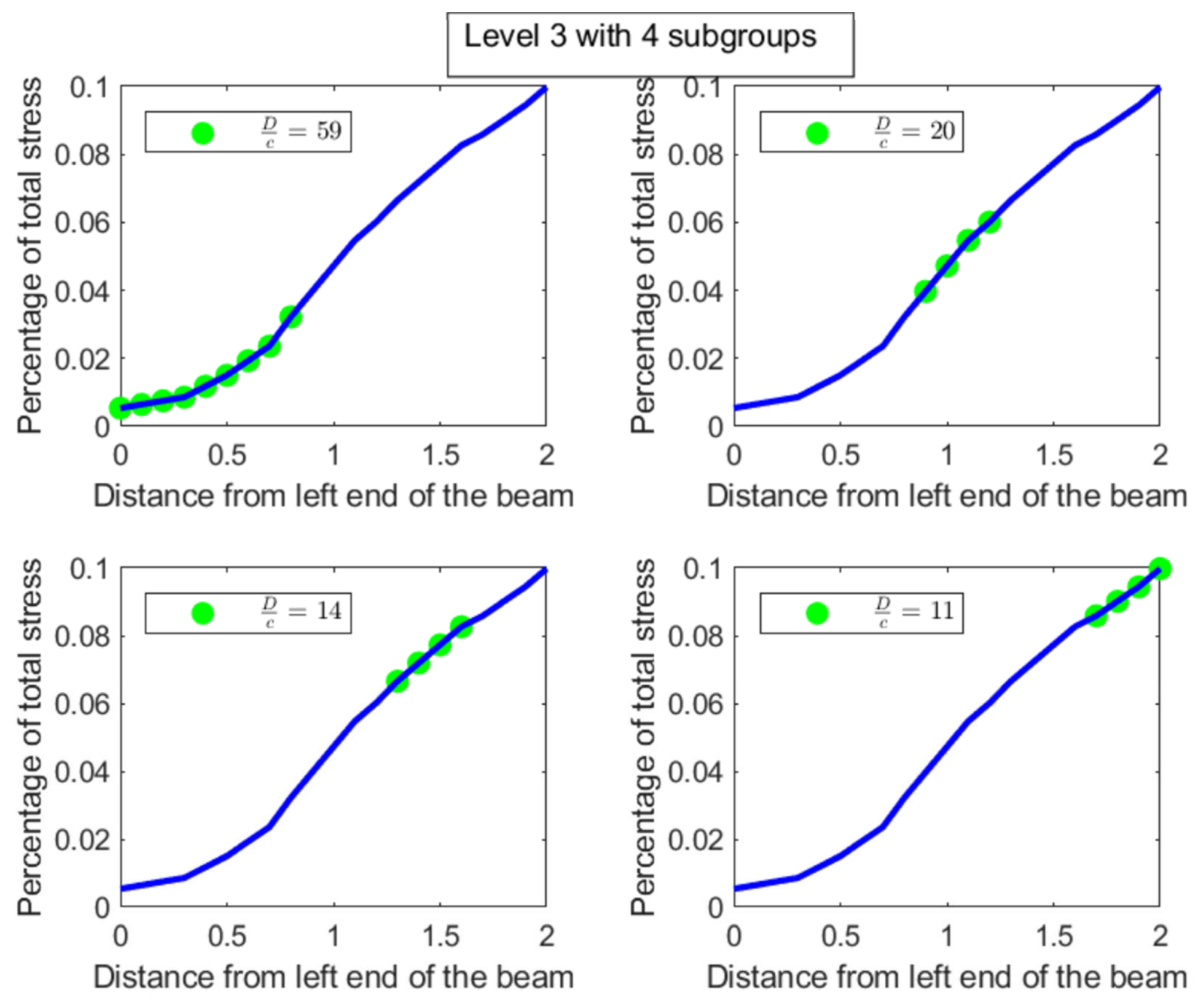
The parts in Level 4 of the stress on a beam tree shown as green dots overlayed on the original distribution shown as a blue line.

Like in the disease contraction example, we could discuss all the sub-parts across all levels, but such a discussion would be too tedious. Hence, we choose to discuss levels 2 and 3 from Figure 14 and Figure 15 below:

1. **Level 2:** This is the first division of the entire distribution. There are two sub-parts in this level as stated before, namely [0,1.2] meters and [1.3,2] meters. The [0,1.2] meter part ($\frac{D}{c}=31$) is around 2.5 times more uniformly distributed than the [1.3,2] meter part ($\frac{D}{c}=12$). Alternatively, the [1.3,2] meter part is 2.5 times as heavily concentrated with stress compared to the [0,1.2] part, entropically speaking. The [1.3,2] meter part clearly has the bulk of the stress. Both these parts are shown in Figure 16, with the green dots denoting the sub-parts overlayed on the blue line which is the original distribution.
2. **Level 3:** In level 3, the [0,1.2] meter part from level 2 splits into two sub-parts, namely [0,0.8] meters with a degree of uniformity $\frac{D}{c}=59$ and [0.9,1.2] meters with a degree of uniformity $\frac{D}{c}=20$. Hence, the [0.9,1.2] meter part is almost 3 times more heavily stressed than the [0,0.8] meter part. These two sub-parts are shown in the top-left and top-right pictures in Figure 17. Similarly, the [1.3,2] meter part from level 2 splits into two parts, namely [1.3,1.6] meters with $\frac{D}{c}=14$ and [1.7,2] meters with $\frac{D}{c}=11$, showing that these two parts are more or less equally distributed in their stress load. However, it is interesting to note that both these parts, [1.3,1.6] and [1.7,2] meters, are at least 4 times more concentrated than the [0,0.8] part in their stress load. These two sub-parts in level 3 are shown in the bottom-left and bottom-right pictures of Figure 17. The material composition of the beam should be designed to withstand such inequalities in stress concentration in the parts described with higher stress loads. Similar analysis and comparison of stress loads can be done at a higher level of granularity by looking at levels 4 through 6 if needed by the designer.

We end by noting that the the inequality in distribution of stress across the 2 m beam has been quantified and located. We reiterate that, like in the previous disease contraction example, the monotonicity of degree of uniformity is maintained across the levels in the tree. Furthermore, we could have started the process with any sub-part of the original distribution (not necessarily contiguous) and not just the entire distribution.

### 4.5. Null Set Example

In the third example, we explore the possibility of bona fide null sets for the degree of uniformity measure $m_{D}\left(P\right)$. We first note that the $m_{D}\left(I\right)=0$, i.e., the degree of uniformity of the entire distribution is zero with respect to the diversity *D*. This is because the entire distribution (denoted by the index set *I*) has diversity equal to *D*. We are not interested in this type of a set because *I* is actually not a null set per se, since there can be subsets that have higher and lower degrees of uniformity in general. We explore the possibility of subsets of *I* (i.e., sub-parts of the original distribution) that are null sets, i.e., sets that satisfy the property that all measurable subsets satisfy $m_{D}\left(P\right)=0$. Our main goal in this example is to explore the relationships between the probabilities of parts of P,Z and *N* that need to be satisfied to guarantee the existence of a proper subset Z⊂I that is a null set. With this in mind, we set the stage for our exploration as follows:

1. Let *I* denote the full set of indices in the given distribution, i.e., $D_{I}=D$.
2. Let *P*, *Z* and *N* denote the sub-parts in the next level which are more, equally and less uniformly distributed respectively compared to the entire distribution *I*.
3. Let *D* denote the degree of uniformity of the entire distribution. This also means that the degree of uniformity of *Z* is also equal to *D*, i.e., $\frac{D_{Z}}{c_{Z}}=D$. We also have that $\frac{D_{P}}{c_{P}}>D$. Let $\eta_{1}>1$ be such that $D_{P}=D\eta_{1}>D$. Similarly let $\eta_{2}<1$ be such that $\frac{D_{N}}{c_{N}}=D\eta_{2}<D$. We also have that $p_{i}<\frac{1}{D}\;\forall i\in P$, $p_{i}=\frac{1}{D}\;\forall i\in Z$ and $p_{i}>\frac{1}{D}\;\forall i\in N$ by construction.
4. Let $c_{P},c_{Z}$ and $c_{N}$ be the probabilities of P,Z and *N* respectively. Hence $c_{P}+c_{Z}+c_{N}=1$.

With the above setup in mind, we first note the following from Theorem 2:

(18) $$\begin{array}{ccc} D & = & {\left(\frac{D_{P}}{c_{P}}\right)}^{c_{P}}{\left(\frac{D_{Z}}{c_{Z}}\right)}^{c_{Z}}{\left(\frac{D_{N}}{c_{N}}\right)}^{c_{N}}={\left(D\eta_{1}\right)}^{c_{P}}{\left(D\right)}^{c_{Z}}{\left(D\eta_{2}\right)}^{c_{N}} \end{array}$$

(19) $$\begin{array}{ccc} & & \Rightarrow {\left(\eta_{1}\right)}^{c_{P}}{\left(\eta_{2}\right)}^{c_{N}}=1\Rightarrow \ln\left(\eta_{1}\right)c_{P}+\ln\left(\eta_{2}\right)c_{N}=0 \end{array}$$

(20) $$\begin{array}{ccc} & & c_{P}+c_{N}=1-c_{Z} \end{array}$$

Equations (19) and (20) can be solved simultaneously to give the following relationship between $c_{P},c_{Z}$ and $c_{N}$:

(21) $$c_{P}=\frac{\ln\eta_{1}\left(1-c_{Z}\right)}{\left(\ln\eta_{1}-\ln\eta_{2}\right)};c_{N}=\frac{-\ln\eta_{2}\left(1-c_{Z}\right)}{\left(\ln\eta_{1}-\ln\eta_{2}\right)}.$$

We can now try to choose values for $\eta_{1},\eta_{2}$ and $c_{Z}$ and try to compute $c_{P}$ and $c_{N}$. With those in hand, we can try to create sub-parts P,Z and *N*. To keep things simple, we choose $D=10,c_{Z}=0.6,\eta_{1}=2$ and $\eta_{2}=\frac{1}{2}$. This gives $c_{P}=c_{N}=0.2$.

**Figure 18:**
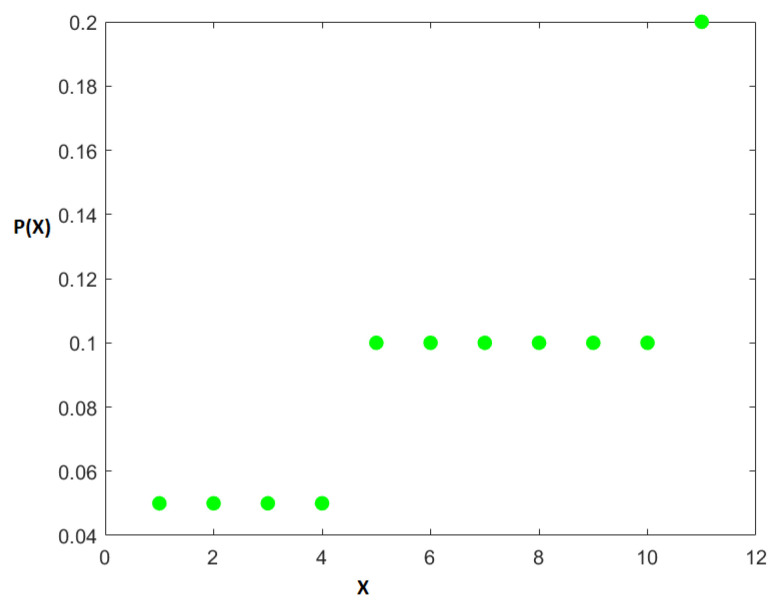
Original distribution consisting of three different uniform subsets for the null set example. The x-axis is an arbitrary random variable *X* and the y-axis is its probability P(X).

1. **Discrete null set:** We choose 6 indices for *Z*, say Z={5,6,7,8,9,10} and place a probability of $p_{i}=\frac{1}{10}\;\forall i\in Z$ for each of those values of *Z*. This means that $\frac{D_{Z}}{c_{Z}}=1/0.1=10=D$, i.e., the degree of uniformity of *Z* is the same as for the entire distribution, thereby confirming that *Z* is indeed a null set. This gives $c_{Z}=6*0.1=0.6$ as needed. Since we need $p_{i}<0.1\;\forall i\in P$, we choose $p_{i}=0.1/2=0.05\;\forall i\in P$. This automatically gives us $\frac{D_{P}}{c_{P}}=1/0.05=20$. And hence $\eta_{1}=2$ is satisfied automatically. We let P={1,2,3,4}. This means $c_{P}=4*0.05=0.2$ as needed. Lastly, we let N={11} and $p_{11}=0.2$. That automatically gives $c_{N}=0.2$ and $\frac{D_{N}}{c_{N}}=1/0.2=5$. Hence $\eta_{2}=\frac{1}{2}$ as needed as well. Hence, putting P,Z and *N* together we have I=P∪Z∪N={1,2,…,11}. Figure 18 shows this entire distribution with green dots for probabilities.

**Figure 19:**
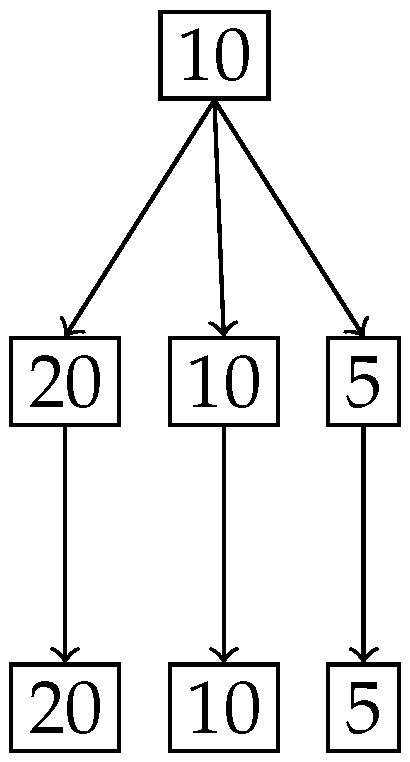
Tree diagram obtained from the nested Hahn decomposition of the Null set example showing the degrees of uniformity, where the middle branch is the non-trivial null set.

**Figure 20:**
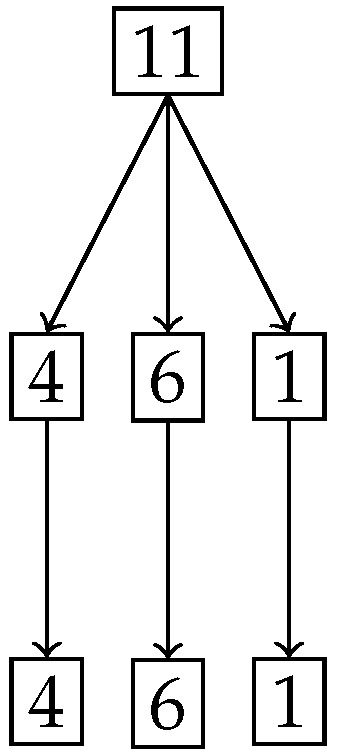
Tree diagram obtained from the nested Hahn decomposition of the null set example where the number of elements of the parts are shown in the form of numbers at each node.

Figure 19 shows the splitting of the degree of uniformity and Figure 20 shows the number of elements in the split. It is clear from Figure 19 that we have a ternary split and the middle portion *Z* has the same degree of uniformity 10 as the entire distribution.

**Figure 21:**
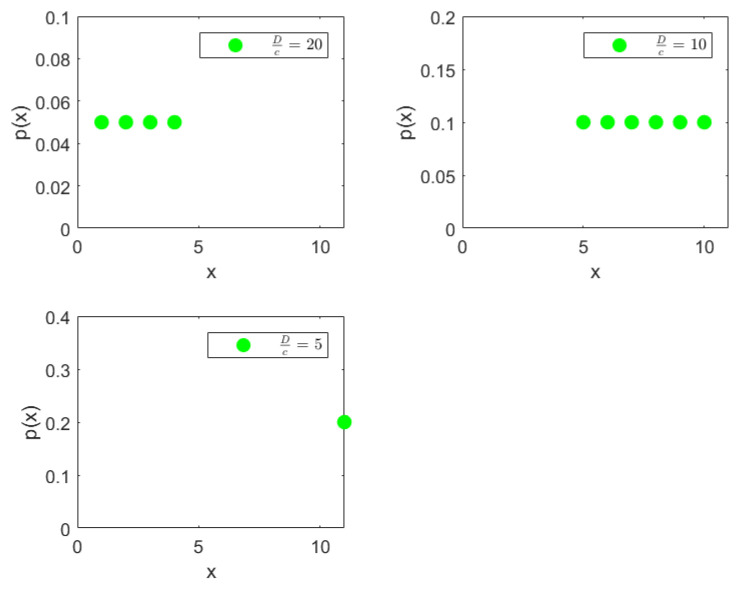
The three levels obtained in the null set. The top right figure with $\frac{D}{c}=10$ is the null set.

Finally Figure 21 shows the three actual parts that are obtained after the ternary split shown as green dots for the probabilities, with the degree of uniformity of parts ordered from left to right across rows. The positive part with $D_{P}=20$ is on the top-left, the null set with $D_{Z}=10$ is on the top-right and the negative set with $D_{N}=5$ is on the bottom-left of Figure 21.

2. **Continuous null set:** We can easily modify the discrete null set example to a continuous one with a density p(x) as follows:
  (22) $$p\left(x\right)=\left\{\begin{array}{cc} 0.05 & 0<x<4\\ 0.1 & 4<x<10\\ 0.2 & 10<x<11 \end{array}\right.$$
  Just like in the discrete null set example, it can be easily shown that Z=(4,10) is a null set, P=(0,4) is a positive set and N=(10,11) is a negative set with respect to the measure $m_{10}$. It can also be easily shown that the diversity of the entire sample space I=(0,11) is given by $D_{I}=10$, $\frac{D_{P}}{c_{P}}=20,\frac{D_{Z}}{c_{Z}}=10$, and $\frac{D_{N}}{c_{N}}=5$ just like in the discrete null set example.

## 5. Conclusions

In this paper, we presented a novel approach for the localization of inequality within a given probability distribution. Our method leverages the Hahn decomposition applied to the newly introduced degree of uniformity measure $m_{D}$, enabling the systematic partitioning of the probability space into three key subsets: P,N and *Z*. These subsets correspond to regions with higher, equal, and lower degrees of uniformity compared to the original distribution, respectively. By recursively applying the Hahn decomposition, we demonstrated that this process generates a specialized ternary tree structure where the degree of inequality decreases monotonically from left to right within each level.

The following are the salient points of the paper:

1. **Granular Analysis of Inequality:** The recursive decomposition reveals increasingly detailed information about the distribution of inequality. At deeper levels, subsets are refined to pinpoint regions of greater or lesser concentration of probability.
2. **Monotonic Preservation:** We showed that the subdivision method ensures monotonicity in the degree of inequality across subsets. This nested ordering guarantees that measurable subsets inherit the inequality hierarchy from their parent sets, preserving consistency and interpretability.
3. **Explicit Null Sets:** Unlike the traditional Hahn decomposition theorem, which leaves ambiguity regarding null sets, our approach explicitly identifies these sets *Z* and isolates them from positive *P* and negative *N* regions, enhancing analytical clarity. These null sets *Z* have the same degree of uniformity as their parent. This is a unique property of the degree of uniformity measure $m_{D}$.
4. **Versatility Across Domains:** Applications to various fields, including disease distribution analysis and stress distribution in engineering beams, demonstrated the utility of our method. The ability to locate and quantify inequality enables tailored interventions in fields such as health care, manufacturing, and environmental science.
5. **Example Validations:** Through detailed examples, we illustrated how the algorithm effectively identifies regions of concentration and uniformity in distributions. The case studies highlighted the method’s robustness in quantifying relative degrees of inequality and isolating key regions for analysis.

Our findings have significant implications across diverse domains. In economics, the ability to pinpoint localized inequality in income distributions can inform targeted policy interventions. In health care, identifying concentrated areas of disease prevalence enables more focused resource allocation and preventive strategies. Moreover, the application to engineering stress distributions demonstrates how material design can be optimized by understanding stress localization.

Future work can extend this framework to multivariate distributions and dynamic settings where probability distributions evolve over time. Another promising avenue involves exploring the implications of the decomposition in machine learning models for feature importance analysis, where understanding the localization of information can enhance interpretability.

Our approach can be used to improve the GINI index by uncovering localized disparities in income or wealth distribution that a single scalar value often conceals. Using recursive Hahn decomposition and the degree of uniformity measure, we can pinpoint where inequality is most pronounced such as in the middle-income range, offering more targeted insights for policy. This enables more precise interventions like tax adjustments or wage reforms and allows dynamic tracking of inequality over time, providing a deeper, more actionable assessment than the GINI index alone.

What sets this paper apart is its introduction of a recursive Hahn decomposition applied to a novel signed measure—the degree of uniformity derived from Shannon entropy—to systematically localize inequality within probability distributions. Unlike traditional approaches that offer only global summaries of inequality, this method constructs a hierarchical, multi-scale partition of the probability space that preserves the ordering of inequality across all levels. It uniquely identifies not only regions of higher and lower concentration but also explicit null sets where uniformity matches that of the whole distribution—resolving ambiguities inherent in classical Hahn decomposition. This framework provides a principled, interpretable, and scalable tool for dissecting the internal structure of inequality, with broad applicability across domains such as epidemiology, engineering, and economics.

In conclusion, the recursive Hahn decomposition of the degree of uniformity measure provides a systematic and insightful tool for dissecting probability distributions. Its ability to partition distributions while preserving the hierarchical order of inequality makes it a valuable framework for both theoretical research and practical applications. By offering a nuanced view of inequality, this approach enables more precise analyses and decision-making across various domains, from economics to health care and engineering. The capacity to track and quantify changes in inequality over time further enhances its utility, making it possible to monitor the effectiveness of interventions or the evolution of inequality in complex systems. As this research reveals new applications, it holds significant potential for advancing equitable and data-driven optimization strategies across multiple fields.

## Data Availability

No new data were created as part of this research.
